# Stochastic Interventional Vaccine Efficacy and Principal Surrogate Analyses of Antibody Markers as Correlates of Protection against Symptomatic COVID-19 in the COVE mRNA-1273 Trial

**DOI:** 10.3390/v15102029

**Published:** 2023-09-29

**Authors:** Ying Huang, Nima S. Hejazi, Bryan Blette, Lindsay N. Carpp, David Benkeser, David C. Montefiori, Adrian B. McDermott, Youyi Fong, Holly E. Janes, Weiping Deng, Honghong Zhou, Christopher R. Houchens, Karen Martins, Lakshmi Jayashankar, Britta Flach, Bob C. Lin, Sarah O’Connell, Charlene McDanal, Amanda Eaton, Marcella Sarzotti-Kelsoe, Yiwen Lu, Chenchen Yu, Avi Kenny, Marco Carone, Chuong Huynh, Jacqueline Miller, Hana M. El Sahly, Lindsey R. Baden, Lisa A. Jackson, Thomas B. Campbell, Jesse Clark, Michele P. Andrasik, James G. Kublin, Lawrence Corey, Kathleen M. Neuzil, Rolando Pajon, Dean Follmann, Ruben O. Donis, Richard A. Koup, Peter B. Gilbert, on behalf of the Immune Assays, Moderna, Inc., Coronavirus Vaccine Prevention Network (CoVPN)/Coronavirus Efficacy (COVE), United States Government (USG)/CoVPN Biostatistics Teams

**Affiliations:** 1 Vaccine and Infectious Disease Division, Fred Hutchinson Cancer Center, Seattle, WA 98109, USA; yhuang@fredhutch.org (Y.H.); nhejazi@hsph.harvard.edu (N.S.H.); lcarpp@fredhutch.org (L.N.C.); yfong@fredhutch.org (Y.F.); hjanes@fredhutch.org (H.E.J.); ylu2@scharp.org (Y.L.); cyu@scharp.org (C.Y.); mandrasik@fredhutch.org (M.P.A.); jkublin@fredhutch.org (J.G.K.); lcorey@fredhutch.org (L.C.); 2 Public Health Sciences Division, Fred Hutchinson Cancer Center, Seattle, WA 98109, USA; 3 Department of Biostatistics, University of Washington, Seattle, WA 98195, USA; avikenny@uw.edu (A.K.); mcarone@uw.edu (M.C.); 4 Department of Biostatistics, T.H. Chan School of Public Health, Harvard University, Boston, MA 02115, USA; 5 Department of Biostatistics, Epidemiology and Informatics, University of Pennsylvania, Philadelphia, PA 19104, USA; bryan.blette@vumc.org; 6 Department of Biostatistics and Bioinformatics, Rollins School of Public Health, Emory University, Atlanta, GA 30322, USA; benkeser@emory.edu; 7 Department of Surgery, Duke Human Vaccine Institute, Duke University Medical Center, Durham, NC 27710, USA; monte@duke.edu (D.C.M.); charlene.mcdanal@duke.edu (C.M.); amanda.eaton@duke.edu (A.E.); msarzott@duke.edu (M.S.-K.); 8 Vaccine Research Center, National Institute of Allergy and Infectious Diseases, National Institutes of Health, Bethesda, MD 20892, USAbritta_f@yahoo.com (B.F.); bob.lin@nih.gov (B.C.L.); rkoup@mail.nih.gov (R.A.K.); 9 Moderna, Inc., Cambridge, MA 02139, USA; weiping.deng@modernatx.com (W.D.); honghong.zhou@modernatx.com (H.Z.); jacqueline.miller@modernatx.com (J.M.); rolando.pajon@modernatx.com (R.P.); 10 Biomedical Advanced Research and Development Authority, Washington, DC 20201, USA; christopher.houchens@hhs.gov (C.R.H.); lakshmi.jayashankar@hhs.gov (L.J.); chuong.huynh@hhs.gov (C.H.); ruben.donis@hhs.gov (R.O.D.); 11 Department of Molecular Virology and Microbiology, Baylor College of Medicine, Houston, TX 77030, USA; hana.elsahly@bcm.edu; 12 Brigham and Women’s Hospital, Boston, MA 02115, USA; lbaden@bwh.harvard.edu; 13 Kaiser Permanente Washington Health Research Institute, Seattle, WA 98101, USA; lisa.a.jackson@kp.org; 14 Division of Infectious Diseases, University of Colorado Anschutz Medical Campus, Aurora, CO 80045, USA; thomas.campbell@ucdenver.edu; 15 Department of Medicine, Division of Infectious Diseases, David Geffen School of Medicine at UCLA, Los Angeles, CA 90095, USA; jlclark@mednet.ucla.edu; 16 Department of Laboratory Medicine and Pathology, University of Washington, Seattle, WA 98195, USA; 17 Center for Vaccine Development and Global Health, University of Maryland School of Medicine, Baltimore, MD 21201, USA; kneuzil@som.umaryland.edu; 18 Biostatistics Research Branch, National Institute of Allergy and Infectious Diseases, National Institutes of Health, Bethesda, MD 20892, USA; dfollmann@niaid.nih.gov

**Keywords:** binding antibody assay, immune correlates of protection, modified treatment policy, neutralizing antibody assay, principal stratification, principal surrogate, SARS-CoV-2, stochastic intervention, stochastic interventional vaccine efficacy

## Abstract

The COVE trial randomized participants to receive two doses of mRNA-1273 vaccine or placebo on Days 1 and 29 (D1, D29). Anti-SARS-CoV-2 Spike IgG binding antibodies (bAbs), anti-receptor binding domain IgG bAbs, 50% inhibitory dilution neutralizing antibody (nAb) titers, and 80% inhibitory dilution nAb titers were measured at D29 and D57. We assessed these markers as correlates of protection (CoPs) against COVID-19 using stochastic interventional vaccine efficacy (SVE) analysis and principal surrogate (PS) analysis, frameworks not used in our previous COVE immune correlates analyses. By SVE analysis, hypothetical shifts of the D57 Spike IgG distribution from a geometric mean concentration (GMC) of 2737 binding antibody units (BAU)/mL (estimated vaccine efficacy (VE): 92.9% (95% CI: 91.7%, 93.9%)) to 274 BAU/mL or to 27,368 BAU/mL resulted in an overall estimated VE of 84.2% (79.0%, 88.1%) and 97.6% (97.4%, 97.7%), respectively. By binary marker PS analysis of Low and High subgroups (cut-point: 2094 BAU/mL), the ignorance interval (IGI) and estimated uncertainty interval (EUI) for VE were [85%, 90%] and (78%, 93%) for Low compared to [95%, 96%] and (92%, 97%) for High. By continuous marker PS analysis, the IGI and 95% EUI for VE at the 2.5th percentile (519.4 BAU/mL) vs. at the 97.5th percentile (9262.9 BAU/mL) of D57 Spike IgG concentration were [92.6%, 93.4%] and (89.2%, 95.7%) vs. [94.3%, 94.6%] and (89.7%, 97.0%). Results were similar for other D29 and D57 markers. Thus, the SVE and PS analyses additionally support all four markers at both time points as CoPs.

## 1. Introduction

In the coronavirus efficacy (COVE) phase 3 clinical trial of the mRNA-1273 COVID-19 vaccine, participants were randomized 1:1 to receive mRNA-1273 vaccine (*n* = 15,209 assigned) or placebo (*n* = 15,206 assigned), administered on Day 1 (D1) and Day 29 (D29) [1,2]. Estimated vaccine efficacy (VE) in baseline negative per-protocol participants against the primary endpoint of virologically confirmed, symptomatic COVID-19 (hereafter, “COVID-19”) starting ≥14 days post-D29 through a median follow-up of 5.3 months, corresponding to completion of the blinded phase, was 93.2% (95% confidence interval (CI), 91.0% to 94.8%) [2]. Vaccine safety was also assessed, with no safety concerns identified during the trial [1,2]. As part of the United States government (USG)-coordinated effort to identify a correlate of protection (CoP) for COVID-19 vaccines [3], we developed a “master protocol” Statistical Analysis Plan (SAP) for harmonizing immune correlates analyses across all of the USG/COVID-19 Response Team phase 3 COVID-19 vaccine trials [4]. As obtaining evidence from multiple analysis frameworks is typically needed to establish an immunologic biomarker for applications such as regulatory decisions or immunobridging, the SAP laid out multiple correlate of risk (CoR) and CoP objectives, each of which addresses a different question. 

In Gilbert et al. [5], we reported the COVE trial results for some of these immune correlate objectives, for the immune markers IgG binding antibodies (bAbs) against the SARS-CoV-2 Spike protein (Spike IgG), IgG bAbs against the Spike receptor binding domain (RBD IgG), 50% inhibitory dilution pseudovirus neutralizing antibody titer (nAb-ID50), and 80% inhibitory dilution pseudovirus neutralizing antibody titer (nAb-ID80), measured on D29 and on D57 in all vaccine recipient breakthrough cases and a randomly sampled immunogenicity subcohort. The IgG markers were measured against the original index strain and nAb-ID50 against the B.1/B.1.2 lineage (NC_045512.2), which is the index strain except with the D614G mutation. All four D57 antibody markers correlated inversely with COVID-19 and impacted mRNA-1273 VE against COVID-19 through ~4 months post-D29 [5]. Similar results were obtained for the D29 antibody markers. In additional analyses of COVE, nAb-ID50 was the strongest independent correlate of risk as determined by machine learning analyses that evaluated multivariable correlates of risk (CoRs) [6]. However, these studies did not report on all the immune correlate objectives outlined in the master protocol SAP. In particular, the assessment of CoPs was based on the controlled VE framework [7] and the natural direct and indirect effects mediation framework [8]. The former framework considers a hypothetical intervention that assigns all participants to receive a vaccine and to have a specific immune marker value, estimates COVID-19 risk under this intervention, and then estimates VE by contrasting this risk with COVID-19 risk under the hypothetical intervention that assigns all participants to receive placebo. The latter framework estimates the proportion of VE that is causally mediated through the immune marker, defined by comparing the natural direct effect to the overall VE.

Here, we report the results from additional CoP analyses of the COVE trial, completing the suite of pre-specified blinded-phase immune correlates analyses of the D29 and D57 antibody markers and COVID-19. Specifically, we evaluated each of the four markers, Spike IgG, RBD IgG, nAb-ID50, and nAb-ID80, measured at D29 or D57, as a CoP against COVID-19 defined using two additional causal inference frameworks for CoP assessment as specified in the master protocol SAP: the stochastic interventional vaccine efficacy (SVE) framework and principal surrogate (PS) framework (within the principal stratification framework of causal inference [9]). The SVE approach, like the controlled VE approach, is based on a hypothetical intervention to modify the immune marker but considers the more plausible stochastic intervention of shifting each vaccine recipient’s immune marker value by a fixed amount relative to their observed marker value instead of the static intervention of deterministically setting the marker level to the same fixed value for all vaccine recipients; thus, the SVE approach defines a contrast relative to the observed marker values, which may plausibly arise in future hypothetical scenarios. The PS approach, in contrast, does not intervene on the immune marker and instead estimates how VE varies across subgroups defined by the value of the immune marker of vaccine recipients (which is a counterfactual variable for participants in the placebo arm). Table 1 summarizes the four statistical frameworks for assessing a correlate of protection from a vaccine efficacy trial.

## 2. Materials and Methods

### 2.1. COVE Trial and Study Endpoint

The COVE trial (NCT04470427), conducted in the United States, enrolled 30,420 adults age 18 and over at appreciable risk of SARS-CoV-2 infection and/or high risk of severe COVID-19 disease and randomly assigned them in a 1:1 ratio to receive either vaccine or placebo [1,2]. The study endpoint used in the correlates analysis, which we refer to as “COVID-19”, is the first occurrence of acute symptomatic COVID-19 with virologically-confirmed SARS-CoV-2 infection [1,2]. Virological confirmation refers to a positive SARS-CoV-2 reverse-transcriptase–polymerase-chain-reaction assay of a nasopharyngeal swab, nasal, or saliva sample. As in Gilbert et al. [5], COVID-19 endpoints beginning seven days post-D29 or -D57, depending on whether D29 or D57 markers were assessed, respectively, through completion of the blinded phase of follow-up were included in the correlates analyses. The calendar dates of this timeframe were 27 July 2020 to 26 March 2021. 

### 2.2. Ethics Statement

All study participants provided written informed consent before enrollment and the protocol and consent forms were approved by the central institutional review board.

### 2.3. Case-Cohort Sets Included in the Correlates Analyses

A case-cohort sampling design [12] detailed in Gilbert et al. [5] was used to sample participants randomly for measurement of antibody markers on D1, D29, and D57. In all vaccine recipients with a breakthrough COVID-19 endpoint, antibody markers were also measured on the same days. As defined in previous studies [1,2], correlates analyses were conducted in baseline negative (no immunologic or virologic evidence of prior COVID-19 at enrollment) per-protocol (received both doses without major protocol violations) participants. The analysis cohort included a randomly sampled immunogenicity subcohort of size 1010 from the vaccine arm plus all vaccine cases starting 7 days after D29 (for D29 correlates analysis) or starting 7 days after D57 (for D57 correlates analysis). The analysis cohort also included all baseline negative per-protocol placebo recipients, without making use of any antibody data because all immune marker values are constant at “zero” (below assay detection or quantitation limits). See Figure S2 in Gilbert et al. [5] for a schematic of participant flow from enrollment through to the analysis. The numbers of vaccine arm cases and non-cases with measured antibody marker data (for each of the four antibody markers) included in the D29 correlates analyses and in the D57 correlates analyses are provided in Table S3. There are 1005 vaccine arm non-cases and 46 (36) vaccine arm cases with D29 (D57) antibody marker data and, hence, they were included for D29 (D57) correlates analyses. All baseline negative per-protocol placebo recipients were included in the analysis (13,221 non-cases and 751 (659) cases). 

### 2.4. Pseudovirus Neutralizing Antibody Assay

Serum nAb activity against SARS-CoV-2 was measured in a validated assay utilizing lentiviral vector pseudotyped with full-length Spike of the D614G strain NC_045512.2 [13]. Assay readouts were calibrated to the World Health Organization 20/136 anti-SARS-CoV-2 immunoglobulin International Standard [14] and are expressed in international units (IU50/mL and IU80/mL for nAb-ID50 and nAb-ID80, respectively). The arithmetic mean calibration factors used to convert assay readouts to international units are provided in Table S4. Table S5 gives the assay limits, with limit of detection (LOD) 2.42 IU50/mL for nAb-ID50 and 15.02 IU80/mL for nAb-ID80. Values below the LOD were assigned the value of LOD/2.

### 2.5. Binding Antibody Assay

Serum IgG bAbs against Spike and RBD were measured using a validated solid-phase electrochemiluminescence S-binding IgG immunoassay [5]. Assay readouts were converted to binding antibody units per ml (BAU/mL) using the World Health Organization 20/136 anti-SARS-CoV-2 immunoglobulin International Standard [14]. Table S5 gives the assay limits, with LOD = 0.3076 BAU/mL for Spike and LOD = 1.593648 for RBD. Values below the LOD were assigned the value of LOD/2.

### 2.6. Stochastic Interventional VE

For each antibody marker, measured levels were hypothetically shifted along a grid, (−1.0, −0.8, −0.6, −0.4, −0.2, 0, 0.2, 0.4, 0.6, 0.8, 1.0), on the log_10_ scale such that −1.0 represents a 10-fold decrease in geometric mean and 1.0 represents a 10-fold increase in geometric mean. For each shift, the average risk of COVID-19 in per-protocol baseline negative vaccine recipients was estimated via the method of Hejazi et al. [10]. Downward (negative) shifts that would result in more than 10% of participants having counterfactual values of the marker below the assay’s LOD were omitted. The zero-shift value corresponds to the observed log_10_ geometric mean marker level. As described in the SAP, these average risk estimates can be translated to the VE scale by also estimating the average risk of per-protocol baseline negative placebo recipients, which are the results that are presented. The analyses were implemented with the txshift and sl3 packages [15,16,17] for the R language and environment for statistical computing [18,19]. 

### 2.7. Binary Principal Surrogate Evaluation

For each antibody marker, the method of Gilbert et al. [20] was used to estimate VE for two principal strata, defined by the immune marker in vaccine recipients being above vs. below the median marker value, with parameters of interest denoted VE(1) = VE(High) and VE(0) = VE(Low). That is, let Y(1) and Y(0) be potential outcomes indicating whether COVID-19 occurs during follow-up if assigned vaccine or placebo, respectively. Let S(1) indicate the marker value at the time point of interest (D29 or D57) if assigned vaccine and let sc indicate a specified cut-point value for S(1) (i.e., median value). Then, the causal estimands of interest are VE(1), VE(0), and RR ratio = (1 − VE(0))/(1 − VE(1)), with

VE(1) = 1 − P(Y(1) = 1|S(1) > sc)/P(Y(0) = 1|S(1) > sc) and

VE(0) = 1 − P(Y(1) = 1|S(1) ≤ sc)/P(Y(0) = 1|S(1) ≤ sc),

where all the probabilities also condition on not experiencing the COVID-19 endpoint by the marker time point of interest (D29 or D57) under both randomization assignments [20]. The relative VE parameter (relative risk ratio = RR ratio) is the degree to which the vaccine confers greater risk reduction for the High subgroup compared to the Low subgroup. The Supplementary Text lists the assumptions needed for the method, which include No Early Harm (NEH) [20], i.e., there are no individuals who would have a COVID-19 outcome before the marker was measured under assignment to vaccine, but not under assignment to placebo. This method relies on user specification of three sensitivity parameters, β_2_, β_3_, and β_4,_ to construct IGI and EUI bounds. These parameters reflect different types and degrees of post-randomization selection bias, all with a log-odds ratio scale, with details in the Supplementary Text and in Gilbert et al. [20]. For data analysis, first, each sensitivity parameter was set to zero, such that the VE parameters were point-identified, and point estimates and 95% CIs were calculated. Then, each of the sensitivity parameters were set to vary from log(0.75) to −log(0.75) (medium robustness) and from log(0.5) to −log(0.5) (high robustness), such that the estimands were partially identifiable, and IGIs and 95% EUIs were calculated.

Table S6 provides the median cut-points for each of the D29 and D57 antibody markers. The method was implemented using the psbinary R package [21].

### 2.8. Continuous Marker Principal Surrogate Evaluation

For each antibody marker measured on a continuous scale, the methods of Huang, Zhuang, and Gilbert [22] were used to estimate the VE curve, i.e., the curve of VE for the subgroup of vaccine recipients with immune markers at each specific level *s*, i.e., VE(*s*) = 1 − P(Y(1) = 1|S(1) = *s*)/P(Y(0) = 1|S(1) = *s*). Specifically, under the NEH assumption, a participant disease-free (i.e., COVID-19 endpoint-free) at the time of marker measurement can belong to either one of the two strata: the “equal-always-at-risk” stratum (where the participant will be disease-free at the time of marker measurement if assigned placebo) or the “early-protected” stratum (where the participant will have experienced the disease outcome by the time of marker measurement if assigned placebo). A model for the mixing probability of the two strata among all vaccine recipients disease-free at the time of marker measurement is assumed with a sensitivity parameter β that equals the log-odds ratio of remaining early-at-risk if assigned placebo for early-at-risk vaccine recipients with Y(1) = 1 relative to Y(1) = 0. Sensitivity analyses were conducted for estimating the VE curve for each marker, with the sensitivity parameter β varying from −log(4) to 0. For comparison, a VE curve estimator was also produced under the EECR assumption, which, in addition to NEH, also assumes that no individuals would have COVID-19 before the marker was measured under placebo assignment but not under vaccine assignment.

## 3. Results

### 3.1. Stochastic Interventional VE Analysis Supports Each of the Four Antibody Markers as a Correlate of Protection

**Figure 1:**
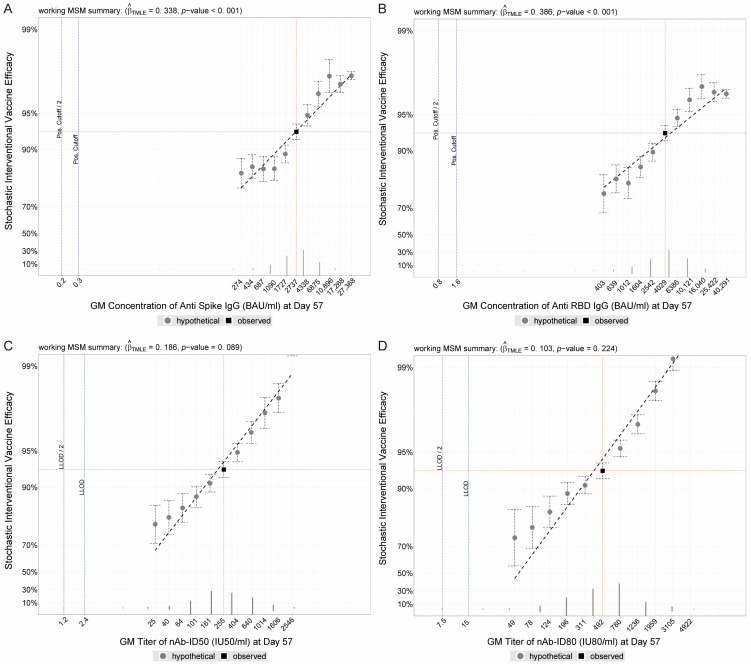
Stochastic interventional vaccine efficacy (SVE) estimates against COVID-19 with hypothetical shifts in geometric mean D57 antibody marker level. SVE, with 95% confidence intervals, for D57 (**A**) Spike IgG, (**B**) RBD IgG, (**C**) nAb-ID50, or (**D**) nAb-ID80, estimated using the method of Hejazi et al. [10]. The y-axis plots the estimated vaccine efficacy (VE) for a vaccine that elicits hypothetical D57 geometric mean value indicated on the x-axis. The vertical red line corresponds to the geometric mean concentration or titer in the COVE study population (baseline negative per-protocol vaccine recipients in the immunogenicity subcohort) and the horizontal red line corresponds to the estimated VE in COVE (follow-up time period from 7 to 100 days post-D57) at a shift of 0, i.e., the observed marker level. BAU, binding antibody units; ID50, 50% inhibitory dilution; ID80, 80% inhibitory dilution; IU, international units; LLOD, lower limit of detection; nAb, neutralizing antibody.

For a given immune marker measured at a post-vaccination time point, SVE analysis [10] posits hypothetical individual-level shifts of the marker’s observed values by different, user-specified magnitudes [10]. The output of SVE analysis is that of the overall VE estimates had the vaccine hypothetically elicited marker values increased (or decreased) as specified by different shift magnitudes. Applying this framework to COVE, estimated mRNA-1273 VE against COVID-19 increased as each D57 marker was hypothetically increased, and vice versa for hypothetical decreases (Figure 1). For example, the observed geometric mean D57 Spike IgG concentration in vaccine recipients was 2737 BAU/mL and the actual cumulative VE from 7 to 100 days post-D57 was 92.9% (95% CI: 91.4%, 93.9%). Overall estimated VE decreased to 84.2% (95% CI: 79.0%, 88.1%) when shifting D57 Spike IgG marker values down one log_10_ (to a geometric mean of 274 BAU/mL), and increased to 97.6% (97.4%, 97.7%) when shifting D57 Spike IgG marker values up one log_10_ (to a geometric mean of 27,368 BAU/mL) (Figure 1A). Results were similar for each of the other three D57 markers.

Similar results were obtained for the D29 binding antibody markers (Figure S1A,B), with the D29 neutralizing antibody markers having less-clear trends with marker GM shifts (Figure S1C,D). Figure S2 (D57 antibody markers) and Figure S3 (D29 antibody markers) show estimates of absolute COVID-19 risk of vaccine recipients under the different shift magnitudes, which are simply the numerators of the estimates of stochastic interventional vaccine efficacy.

### 3.2. Binary Principal Surrogate Analysis Supports Each of the Four Antibody Markers as a Correlate of Protection

**Figure 2:**
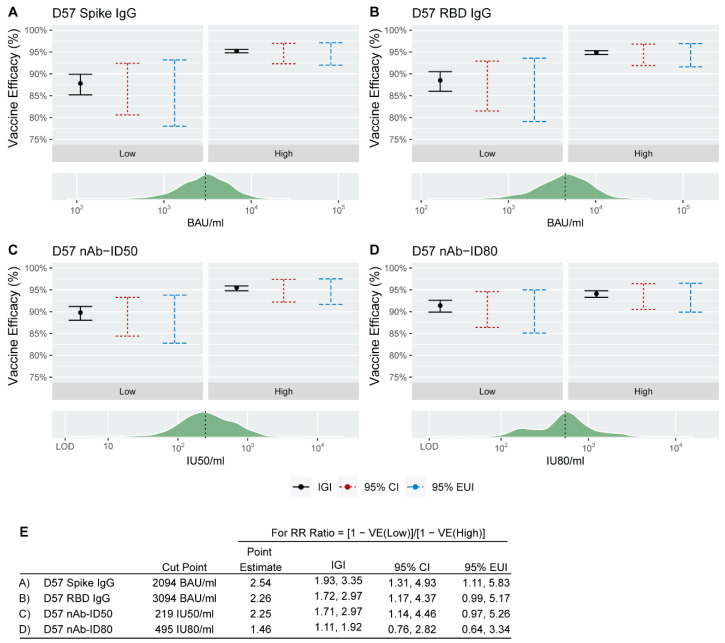
Binary principal surrogate vaccine efficacy (VE) against COVID-19 by D57 antibody marker greater than vs. less than or equal to the designated cut-point (median value). The black dot in each panel corresponds to the VE estimate for the relevant D57 antibody marker subgroup (Low or High) for (**A**) Spike IgG, (**B**) RBD IgG, (**C**) nAb-ID50, or (**D**) nAb-ID80 when β sensitivity parameters are set to zero. The vertical black line denotes the ignorance interval (IGI) when β sensitivity parameters range from log(0.75) to −log(0.75), the vertical red dashed line denotes the 95% confidence interval (CI) when β sensitivity parameters are set to zero, and the vertical blue dashed line denotes the 95% estimated uncertainty interval (EUI) when β sensitivity parameters range from log(0.75) to −log(0.75). The green histogram on each lower panel denotes the distribution of the D57 antibody marker, with the vertical black dashed line placed at the cut-point separating a Low D57 antibody marker response from a High D57 antibody marker response. This cut-point was the median marker value in baseline negative per-protocol vaccine recipients in the immunogenicity subcohort. (**E**) For each antibody marker, cut-point, relative risk (RR) ratio point estimate, IGI, 95% CI, and 95% EUI. RR ratio = (1 − VE(0))/(1 − VE(1)). BAU, binding antibody units; ID50, 50% inhibitory dilution; ID80, 80% inhibitory dilution; IU, international units; nAb, neutralizing antibody.

For a given immune marker measured at a post-vaccination time point, PS analysis estimates VE for each of two or many subgroups defined by a participant’s value of the immune marker if assigned to the vaccine arm (observable in vaccine recipients and a counterfactual in placebo recipients). PS analysis was applied under the assumption that vaccination does not cause an increased risk of COVID-19 prior to the time of immune marker measurement (i.e., the NEH assumption) [20]. First, the approach was applied to estimate VE for each of two subgroups with High or Low immune marker value using the median marker value to define High vs. Low. Results are presented using ignorance intervals (IGIs) and 95% estimated uncertainty intervals (EUIs) [23]. The IGI is a range of VE point estimates, with each estimate calculated under specific fixed values of the sensitivity parameters. A 95% EUI is the union of 95% CIs, where each 95% CI is calculated under specific fixed values of the sensitivity parameters. Whereas 95% CIs only capture uncertainty due to sampling variability, 95% EUIs capture additional uncertainty due to partial non-identifiability of the subgroup VE parameters that occurs because the counterfactual immune marker values if assigned vaccine are not measured for participants who were actually assigned placebo. Results are presented in three ways by specifying three possible ranges for each of three user-specified sensitivity parameters β_2_ = β_3_ = β_4_ that are defined in Section 5.4 of Gilbert et al. [20]: [log(1.0), −log(1.0) = 0, 0], [log(0.75), −log(0.75)], or [log(0.50), −log(0.50)], which specify different types and degrees of post-randomization selection bias (see Methods and Supplementary Text for definitions of the sensitivity parameters). For each of the four immune markers at D57, VE point estimates were greater for the High D57 marker subgroup compared to the Low D57 marker subgroup (Table 2, Figure 2). In the special case of setting β_2_ = β_3_ = β_4_ = 0, IGIs collapse to point estimates and EUIs collapse to CIs, in which case estimated VE (95% CI) for Low vs. High D57 Spike IgG was 88% (81, 92%) vs. 95% (92, 97%), and results for the other three immune markers were similar. To assess whether VE differed between the Low and High subgroups, we also estimated ratios (1 − VE(Low))/(1 − VE(High)). For example, when setting β_2_ = β_3_ = β_4_ = 0, the point estimate (95% CI) for D57 Spike IgG was 2.54 (1.31, 4.93), supporting higher VE for the High marker subgroup. This inference is robust to a moderate amount of allowed uncertainty (95% EUI 1.11, 5.83 when each sensitivity parameter is specified to range over [log(0.75), −log(0.75)]), but not robust to the higher amount of allowed uncertainty (95% EUI 0.72, 9.69 for each sensitivity parameter specified to range over [log(0.50), −log(0.50)]). Similar results were obtained for the four D29 markers (Table S1, Figure S4), with the lower limit of the IGI for the (1 − VE(Low))/(1 − VE(High)) ratio exceeding one under a moderate amount of allowed uncertainty for all four markers.

### 3.3. Continuous Principal Surrogate Analysis Supports Each of the Four Antibody Markers as a Correlate of Protection

**Figure 3:**
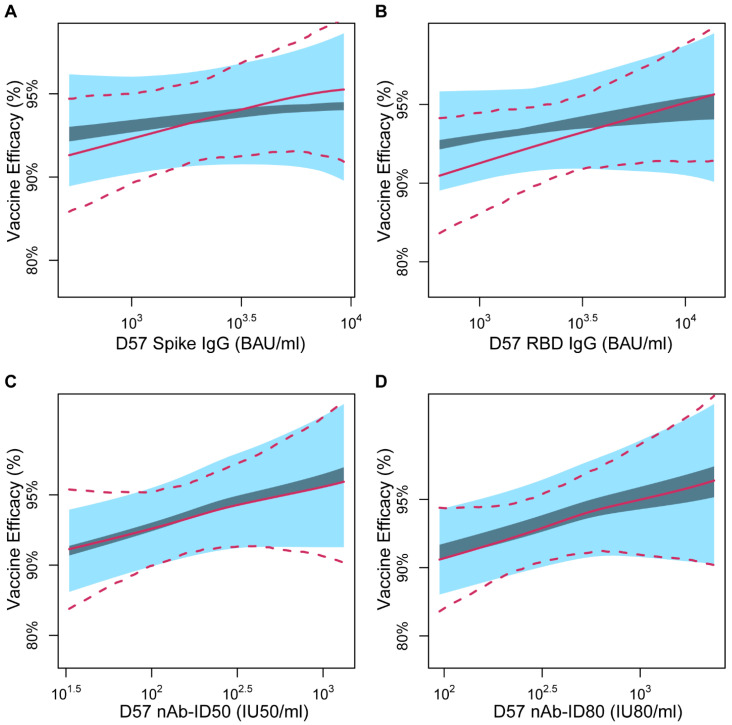
Continuous principal surrogate vaccine efficacy (VE) against COVID-19 by D57 marker level, with ignorance intervals (dark blue) and 95% estimated uncertainty intervals (light blue) under the No Early Harm (NEH) assumption shown for the sensitivity parameter β assumed to fall in the range [−log(4), 0]. Results are shown for (**A**) Spike IgG, (**B**) RBD IgG, (**C**) nAb-ID50, or (**D**) nAb-ID80. In each panel, the solid and dashed lines are the estimated VE curve and 95% perturbation confidence intervals with the Equal Early Clinical Risk (EECR) assumption. The curves are plotted over the marker range of the 2.5th to 97.5th percentile (Spike IgG: 519 to 9263 BAU/mL, RBD IgG: 638 to 13,794 BAU/mL, nAb-ID50: 33 to 1321 IU50/mL, nAb-ID80: 95 to 2385 IU80/mL). BAU, binding antibody units; ID50, 50% inhibitory dilution; ID80, 80% inhibitory dilution; IU, international units; nAb, neutralizing antibody.

Second, the PS method of Huang, Zhuang, and Gilbert [22] was applied to estimate the VE curve that describes how VE varies by subgroups defined by each possible value of a quantitative immune marker if assigned to the vaccine arm. Estimated VE curves for the D57 and D29 markers with *s* values (hereafter “VE(*s*)”) ranging from the 2.5th to 97.5th percentiles are shown in Figure 3 and Figure S5, respectively. Corresponding VE estimates (IGIs and EUIs) are presented in Table 3 and Table S2, respectively. For all four antibody markers and both time points, the IGI for VE(*s*) under each specified value of the sensitivity parameter β varying over the pre-specified range of [−log(4), 0] (see Methods for a definition of β). For all D57 markers, the lower 95% EUI limit for VE(*s*) exceeded 80% for all observed *s* values above its 2.5th percentile. For example, the IGI and EUI for VE(*s*) at the 2.5th vs. 97.5th percentile of *s* were [92.6%, 93.4%] (89.2%, 95.2%) vs. [94.3%, 94.6%] (89.7%, 97%) for D57 Spike IgG, and results were similar for the other three immune markers (Table 3).

As a comparison, in Figure 3 and Figure S5, Table 3 and Table S2, we also present corresponding analyses under the simpler but less realistic Equal Early Clinical Risk (EECR) assumption, under which there is no sensitivity parameter β. EECR assumes no vaccine effect on risk of COVID-19 prior to the time of immune response measurement and is thus not a reasonable assumption given the observed early vaccine effect in the COVE trial [1,2]. For the two binding antibody markers, the results under EECR show greater moderation of VE across the range of marker values than the results under NEH, whereas for the two neutralizing antibody markers, the results are similar. These results are mostly of academic interest given that the NEH assumption is well justified, whereas the EECR assumption is violated. The overall conclusion is that all PS analyses support that VE increases across subgroups with increasing D29 and D57 binding and neutralizing antibody levels.

## 4. Conclusions

Results from the stochastic interventional and principal surrogate statistical frameworks for assessing immune markers as correlates of protection (CoP) supported all four antibody markers measured at D29 and D57 as CoPs for two-dose mRNA-1273 vaccine protection against COVID-19 through the COVE blinded phase with a median follow up of 5.3 months post dose two. These findings add to our previous findings supporting CoPs via the controlled VE and mediation frameworks [7,8], thus adding to the body of evidence characterizing these markers as CoPs.

The SVE results suggested that the D57 neutralizing antibody markers were potentially stronger CoPs than the D57 binding antibody markers. For example, there was a greater increase in VE on the relevant multiplicative scale across the range of hypothetical shifts (from a 10-fold decrease to a 10-fold increase) for D57 nAb-ID80 compared to D57 Spike IgG: For D57 nAb-ID80, estimated VE increased from 74.5% (56.5% to 85.1%) to 99.7% (99.6% to 99.7%) (an 85.0-fold increase in the amount of vaccine protection on the multiplicative scale), whereas for D57 Spike IgG, estimated VE increased from 84.2% (95% CI, 79.0% to 88.1%) to 97.6% (97.4% to 97.7%) from the lowest to the highest shift (a 6.6-fold increase in the amount of vaccine protection on the multiplicative scale). This difference between the D57 neutralizing antibody and D57 binding antibody markers was less pronounced for the continuous marker principal surrogate analyses. For example, the IGIs for VE at the 2.5th and 97.5th percentile of marker values were [90.9%, 92.1%] and [95.1%, 96.4%] for D57 nAb-ID80 compared to [92.6%, 93.4%] and [94.3%, 94.6%] for D57 Spike IgG. Moreover, in the binary principal surrogate analysis, there was about a 2-fold multiplicative-scale increase in the amount of vaccine protection going from the Low to High marker subgroup for both antibody markers.

For the D29 markers, the SVE results indicated a similar change in VE across shifts of binding antibody and neutralizing antibody markers, where only shifts upward can be compared (up to 10-fold higher). For example, for D29 Spike IgG, estimated VE increased from 92.9% (95% CI: 91.4%, 93.9%) at no shift to 97.3% (96.0%, 98.2%) at the highest shift (a 2.6-fold multiplicative-scale increase), whereas for D29 nAb-ID50, these values were 92.6% (91.5% to 93.5%) to 96.3% (93.0% to 98.1%) (a 2.0-fold multiplicative-scale increase). By the binary principal surrogate analysis, the change in VE from Low to High marker subgroups was also similar for binding and neutralizing antibody markers: 88% (81% to 92%) vs. 95% (95% CI: 92% to 96%) for D29 Spike IgG compared to 89% (84% to 92%) vs. 95% (92% to 97%) for D29 nAb-ID80 (at sensitivity parameters set to zero).

While we have presented evidence in multiple papers for D29 and D57 antibody markers as CoPs, these markers do not fully represent the entire repertoire of immune responses that can provide protection and did not show perfect mediation of the vaccine’s efficacy against COVID-19. This can be partly seen in the PS analyses where vaccine efficacy estimates are well above zero at the lowest end of the biomarker level, which implies the average causal necessity condition for a perfect principal surrogate does not hold. It would be interesting to pursue the identification of improved CoPs by studying other immune responses such as Fc effector and T-cell responses. Technical advances for measuring neutralization more sensitively would also be of interest.

This study shares many of the strengths and limitations of our previous COVID-19 vaccine immune correlates studies, including the constrained scope to a study population naïve to SARS-CoV-2 who received two [5,6,24,25] vaccine or placebo doses, i.e., no boosters. Future work is planned to apply the SVE analysis framework to the COVE trial to assess CoPs for both SARS-CoV-2-naïve and -positive populations and for recipients of a third dose. Additionally, during the follow-up period for correlates analysis, the study population was exposed to predominantly ancestral lineage SARS-CoV-2 (i.e., the D614G B.1/B.1.2 strain) and secondarily to minor genetic drift variants [26]. The antibody markers assessed as correlates in this and our previous studies were measured against the index strain (binding antibodies) or the D614G strain (neutralizing antibodies), thus essentially being matched to the exposing D614G strain viruses. Future work is ongoing to assess these markers and counterpart markers measured against the Omicron BA.1 strain as correlates of Omicron BA.1 COVID-19.

The SVE and PS methods have specific strengths and weaknesses distinguishing them from the previously applied controlled VE and mediation methods. SVE analysis has advantages over both previously applied frameworks in that its hypothetical interventions on an immune marker are more conceivable and can be guided by data. For example, data on how a refined vaccine regimen changes the distribution of an antibody marker can be used to empirically specify a marker shift of interest, and SVE analysis applied to estimate how VE would change had the refined vaccine regimen been evaluated in the VE trial. PS analysis, by not involving any hypothetical intervention on an immune marker, advantageously avoids any issues with the conceivability of the causal estimand. However, the absence of an intervention on the marker implies that the PS framework does not provide results that can be interpreted in terms of an immune marker’s causal effect on disease risk (fitting one perspective in causal inference on “no causation without manipulation” [27]); PS analysis is “vanilla subgroup analysis” that obtains separate VE estimates across a range of subgroups. That is, whereas the previously applied SVE CoP frameworks rely on the key assumption that the immune marker is randomized after accounting for other measured participant characteristics, the PS framework does not require this assumption. However, PS analysis replaces this challenge with the equally challenging issue of missing data on the potential immune markers of placebo recipients, which is generally tackled by crossing over placebo recipients to the vaccine arm and measuring their immune markers and/or by measuring pre-vaccination characteristics that predict the post-vaccination immune marker, as well as by specifying pattern mixture models with sensitivity parameters to express the type and degree of post-randomization selection bias.

The PS and SVE methods can be generally applied to any vaccine efficacy trial for which the required immune marker data are available; for instance, the PS framework has been applied to vaccine efficacy trial data for dengue [28], herpes zoster [29], HIV [30], RSV [31], and influenza [32], and the SVE framework has been applied to HIV [10]. Both methods can be readily applied when all participants in the analyzed cohort have no evidence of prior infection with the pathogen of interest, as in the current analysis. Some additional considerations for the PS method are needed if the analyzed cohort includes participants who were previously infected with the pathogen of interest, e.g., the SARS-CoV-2-non-naïve population. In these scenarios, the need to estimate a VE curve is expanded to the need to estimate a VE surface conditional on the pair of potential immune markers under both treatment assignments, to the vaccine and to the placebo [11].

## Figures and Tables

**Table 1:** Four statistical frameworks for assessing an immune marker measured at a post-vaccination time point as an immune correlate of protection (CoP) against a clinical outcome from a vaccine efficacy trial, all of which were applied to the COVE trial.

Statistical Framework for Assessing a CoP	Objective of the CoP Analysis Applied to an Immune Marker in COVE
Controlled vaccine efficacy (VE) [7]	To assess the vaccine efficacy that would be observed under a hypothetical intervention that assigns all participants to the vaccine arm and to a specific value of the marker, as opposed to assigning all participants to placebo *
Mediation of VE [8]	To assess the proportion of the overall VE against COVID-19 that is mediated through the marker, through assessment of the natural direct effect (NDE) of vaccine assignment on COVID-19 (NDE = the component of VE that remains after setting (deactivating) the marker to the level it would have if assigned to the placebo arm)
Stochastic interventional VE [10]	To assess how overall VE would change under user-specified shifts of marker values of vaccine recipients from their observed values
Principal surrogate VE [11]	To assess how VE varies over subgroups defined by the marker value if assigned to the vaccine arm

* This objective/definition attains in studies where all placebo recipients have the same value of the immune marker. This occurs in COVE, as baseline negative placebo recipients all have antibody levels to SARS-CoV-2 antigens that are below the assay detection limit. For VE trials where placebo recipients have variability in the immune marker (i.e., studies that enroll individuals previously infected with the pathogen), the objective/definition must be updated: to assess the VE that would be observed under a hypothetical intervention that assigns all participants to the vaccine arm and to a specific value of the marker, as opposed to assigning all participants to placebo and to a specific value of the marker.

**Table 2:** Principal surrogate correlates of vaccine efficacy (VE) results by Gilbert et al. method [20] for High (above median) vs. Low (below median) D57 antibody marker vaccinated subgroups under the No Early Harm (NEH) assumption with sensitivity analysis scenarios.

		VE(0)		VE(1)		(1 − VE(0))/(1 − VE(1))	
		Low Marker Vaccine Subgroup		High Marker Vaccine Subgroup		Relative Risk Ratio	
Marker	Sens. *	Ignorance Interval	95% Estimated Uncertainty Interval	Ignorance Interval	95% Estimated Uncertainty Interval	Ignorance Interval	95% Estimated Uncertainty Interval
D57 Spike IgG	None	(0.88, 0.88)	(0.81, 0.92)	(0.95, 0.95)	(0.92, 0.97)	(2.54, 2.54)	(1.31, 4.93)
D57 Spike IgG	Med	(0.85, 0.90)	(0.78, 0.93)	(0.95, 0.96)	(0.92, 0.97)	(1.93, 3.35)	(1.11, 5.83)
D57 Spike IgG	High	(0.80, 0.92)	(0.70, 0.95)	(0.94, 0.96)	(0.91, 0.98)	(1.30, 5.08)	(0.72, 9.69)
D57 RBD IgG	None	(0.89, 0.89)	(0.81, 0.93)	(0.95, 0.95)	(0.92, 0.97)	(2.26, 2.26)	(1.17, 4.37)
D57 RBD IgG	Med	(0.86, 0.90)	(0.79, 0.94)	(0.94, 0.95)	(0.92, 0.97)	(1.72, 2.97)	(0.99, 5.17)
D57 RBD IgG	High	(0.81, 0.93)	(0.72, 0.95)	(0.93, 0.96)	(0.90, 0.98)	(1.15, 4.50)	(0.64, 8.48)
D57 nAb-ID50	None	(0.90, 0.90)	(0.84, 0.93)	(0.95, 0.95)	(0.92, 0.97)	(2.25, 2.25)	(1.14, 4.46)
D57 nAb-ID50	Med	(0.88, 0.91)	(0.83, 0.94)	(0.95, 0.96)	(0.92, 0.97)	(1.71, 2.97)	(0.97, 5.26)
D57 nAb-ID50	High	(0.84, 0.93)	(0.78, 0.95)	(0.94, 0.97)	(0.90, 0.98)	(1.15, 4.50)	(0.63, 8.95)
D57 nAb-ID80	None	(0.91, 0.91)	(0.86, 0.95)	(0.94, 0.94)	(0.91, 0.96)	(1.46, 1.46)	(0.76, 2.82)
D57 nAb-ID80	Med	(0.90, 0.93)	(0.85, 0.95)	(0.93, 0.95)	(0.90, 0.97)	(1.11, 1.92)	(0.64, 3.34)
D57 nAb-ID80	High	(0.87, 0.94)	(0.80, 0.96)	(0.92, 0.95)	(0.88, 0.97)	(0.74, 2.90)	(0.41, 5.37)

* Sensitivity parameter settings were: None, β sensitivity parameters β_2_, β_3_, β_4_ set to zero; Med, β sensitivity parameters β_2_, β_3_, β_4_ ranging from log(0.75) to −log(0.75); High, β sensitivity parameters β_2_, β_3_, β_4_ ranging from log(0.5) to −log(0.5).

**Table 3:** Principal surrogate correlates of vaccine efficacy results by the Huang, Zhuang, and Gilbert method [22] for D57 antibody marker at various levels under the No Early Harm (NEH) or Equal Early Clinical Risk (EECR) assumption.

			Vaccine Efficacy (S_alpha)						
Marker	Assumption		Alpha = 0.025	0.05	0.1	0.5	0.9	0.95	0.975
D57 Spike IgG		Concentration (BAU/mL)	519.4	862.1	1224	2926.2	6169.4	7724.8	9262.9
	EECR	Estimate (%)	91.7	92.5	93	94.2	95.0	95.1	95.2
		CI (%)	(86.7, 94.8)	(88.8, 95)	(90.1, 95.1)	(91.5, 96)	(91.9, 96.9)	(91.6, 97.1)	(91.2, 97.4)
	NEH	IGI (%)	[92.6, 93.4]	[93, 93.7]	[93.3, 93.9]	[93.9, 94.3]	[94.2, 94.5]	[94.2, 94.6]	[94.3, 94.6]
		EUI (%)	(89.2, 95.7)	(90.1, 95.7)	(90.5, 95.7)	(91, 96.1)	(90.7, 96.6)	(90.3, 96.8)	(89.7, 97)
D57 RBD IgG		Concentration (BAU/mL)	637.9	1093.5	1670.9	4423.3	9361.8	11,560.8	13,793.5
	EECR	Estimate (%)	90.6	91.8	92.6	94.1	95	95.2	95.4
		CI (%)	(84.4, 94.4)	(87.4, 94.7)	(89.4, 94.8)	(91.4, 95.9)	(91.7, 97)	(91.8, 97.2)	(91.8, 97.5)
	NEH	IGI (%)	[92.6, 93.2]	[93.2, 93.6]	[93.5, 93.9]	[93.9, 94.7]	[94.2, 95.2]	[94.3, 95.4]	[94.3, 95.5]
		EUI (%)	(89.3, 95.5)	(90.4, 95.6)	(90.9, 95.6)	(91.1, 96.4)	(90.7, 97)	(90.5, 97.2)	(90.1, 97.3)
D57 nAb-ID50		Titer (IU50/mL)	33	60.8	88.7	248.1	786.5	1100.8	1320.8
	EECR	Estimate (%)	91.5	92.3	92.9	94.2	95.2	95.5	95.6
		CI (%)	(84.6, 95.3)	(88, 95.1)	(89.5, 95.1)	(91.6, 96)	(91.2, 97.4)	(90.7, 97.8)	(90.3, 98)
	NEH	IGI (%)	[90.9, 91.7]	[92, 92.7]	[92.6, 93.2]	[94.2, 94.7]	[95.2, 95.7]	[95.5, 96]	[95.6, 96.2]
		EUI (%)	(87, 94.2)	(88.7, 94.8)	(89.6, 95.2)	(91.3, 96.4)	(91.6, 97.5)	(91.6, 97.8)	(91.6, 98)
D57 nAb-ID80		Titer (IU80/mL)	94.7	130.6	161.7	544.9	1248.9	1871.8	2385
	EECR	Estimate (%)	90.8	91.5	92	94.3	95.2	95.6	95.9
		CI (%)	(84.4, 94.6)	(86.7, 94.6)	(88, 94.6)	(91.4, 96.2)	(91, 97.4)	(90.7, 97.9)	(90.3, 98.2)
	NEH	IGI (%)	[90.9, 92.1]	[91.5, 92.7]	[91.9, 93]	[94, 94.9]	[94.6, 95.8]	[94.9, 96.2]	[95.1, 96.4]
		EUI (%)	(86.9, 94.5)	(87.8, 94.8)	(88.4, 95.1)	(91, 96.6)	(90.9, 97.5)	(90.6, 97.9)	(90.4, 98.1)

CI, 95% confidence interval; EUI, 95% estimated uncertainty interval; IGI, ignorance interval. alpha = percentile of marker in vaccine recipients.

## Data Availability

Access to patient-level data and supporting clinical documents with qualified external researchers may be available upon request and subject to review.

## Supplementary Materials

The following supporting information can be downloaded at: https://www.mdpi.com/article/10.3390/v15102029/s1.
