# Supplementary material for: Stochastic Interventional Vaccine Efficacy and Principal Surrogate Analyses of Antibody Markers as Correlates of Protection against Symptomatic COVID-19 in the COVE mRNA-1273 Trial
Source: Viruses. 2023 Sep 29;15(10):2029. doi: 10.3390/v15102029 (PMC10612023; doi:10.3390/v15102029)
Supplement: Supplementary file 1 [file viruses-15-02029-s001.zip › viruses-2531818-supplementary.pdf]

Immune Assays Team
(PubMed listed, and ordered alphabetically by affiliation)

Affiliation	Team Members
Biomedical Advanced Research and Development Authority (BARDA), Washington, DC	Oleg Borisov, Flora Castellino, Brett Chromy, Mark Delvecchio, Ruben O. Donis, Tremel Faison, Corey Hoffman, Christopher Houchens, Tom Hu, Chuong Huynh, Pennie Hylton, Lakshmi Jayashankar, Aparna Kolhekar, James Little, Karen Martins, Jeanne Novak, Carol Sabourin, Evan Sturtevant, Xiaomi Tong, John Treanor, Danielle Turley, Leah Watson
Boston Consulting Group, Boston, MA	Gian King, Andrew Li, Najaf Shah, Smruthi Suryaprakash, Jue Xiang Wang
Division of AIDS, NIAID, NIH, Bethesda, MD	Patricia D'Souza
Division of MID (Microbiology and Infectious Diseases), NIAID, NIH, Bethesda, MD	Janie Russell
Duke University, Durham, NC	David Beaumont, Kendall Bradley, Jiayu Chen, Xiaoju Daniell, Thomas Denny, Elizabeth Domin, Amanda Eaton, Kelsey Engel, Wenhong Feng, Juanfei Gao, Hongmei Gao, Kelli Greene, Sarah Hiles, Leihua Liu, Kristy Long, Kellen Lund, Charlene McDanal, David C. Montefiori, Marcella Sarzotti-Kelsoe, Francesca Suman, Haili Tang, Jin Tong, Olivia Widman
The Tauri Group, an LMI company - Contract Support for U.S. Department of Defense (DOD) Joint Program Executive Office for Chemical, Biological, Radiological and Nuclear Defense (JPEO-CBRND) Joint Project Manager for Chemical, Biological, Radiological, and Nuclear Medical (JPM CBRN Medical), Fort Detrick, Maryland, USA	Christopher S. Badorrek, Gregory E. Rutkowski
Vaccine Research Center, NIAID, NIH, Bethesda, MD	Akua Abrah, Obrimpong Amoa-Awua, Manjula Basappa, Robin Carroll, Erykah Coe, Jevone Fentress, Britta Flach, Suprabhath Gajjala, Nazaire Jean-Baptiste, Richard A. Koup, Bob C. Lin, Adrian McDermott, Christopher Moore, Mursal Naisan, Muhammed Naqvi, Sandeep Narpala, Sarah O'Connell, Abhinaya Srikanth, Clare Whittaker, Weiwei Wu

Moderna, Inc. Team
(PubMed listed, ordered alphabetically)

Affiliation	Team Members
Moderna, Inc., Cambridge, MA	Weiping Deng, Shu Hahn, Jacqueline Miller, Rolando Pajon, Honghong Zhou

Coronavirus Vaccine Prevention Network (CoVPN)/Coronavirus Efficacy (COVE) Team
(PubMed listed, and ordered alphabetically by institution affiliation)

Affiliation/Funding*	Study Group	Location
AB Clinical Trials	Atoya Adams, MD, MBA, Eric Miller	Las Vegas, NV
Accel Research Sites	Bruce G. Rankin DO, John Hill MD, Steven Shinn MD, Marshall Nash MD	DeLand, FL
Advanced Clinical Research	Sinikka L. Green MD, Colleen Jacobsen, Jayasree Krishnankutty, Sikhongi Phungwayo	Cedar Park, TX
Alliance for Multispecialty Research	Richard M. Glover, II MD, Drs. Stacy Slechta, Troy Holdeman, Robyn Hartvickson, Amber Grant	Newton, KS
Alliance for Multispecialty Research	Terry L. Poling MD, Terry D. Klein MD, Thomas C. Klein MD, Tracy R. Klein MD	Wichita, KS
Alliance for Multispecialty Research	William B. Smith MD, Richard L. Gibson MD, Jennifer Winbigler MD, Elizabeth Parker PA	Knoxville,TN
Baptist Health Center for Clinical Research	Priyantha N. Wijewardane, MD, Eric Bravo MD, Jeffrey Thessing MD, Michelle Maxwell APRN, Amanda Horn APRN	Little Rock, AR
Baylor College of Medicine, NIAID 1UM1AI148575-01S2	Hana El Sahly MD, Jennifer Whitaker MD, Catherine Mary Healy MD, Christine Akamine MD	Houston, TX
Benchmark Research	Laurence Chu, MD, R. Michelle Chouteau, MD	Austin, TX
Benchmark Research	Michael J. Cotugno MD, George H. Bauer, Jr. MD	Metairie, LA
Benchmark Research	Greg Hachigian MD, Masaru Oshita MD, Michael Cancilla NP, Deborah Murray NP, Kristen Kiersey NP	Sacramento, CA
Benchmark Research	William Seger MD, Mohammed Antwi, Allison Green, Anthony Kim	Fort Worth, TX
Brigham and Women's Hospital, NIAID UM1AI069412, NCATS UL1RR025758	Lindsey R Baden MD, Michael Desjardins MD, Jennifer A Johnson MD, Amy Sherman MD, Stephen R Walsh MD	Boston, MA
Carolina Institute for Clinical Research	Judith Borger DO, Ryan Starr DO, Scott Syndergaard DO, Nafisa Saleem MD	Fayetteville, NC
Centex Studies	Joel Solis MD, Martha Carmen Medina PA-C, Westly Keating PA-C, Edgar Garcia PA-C, Cynthia Bueno PA-C	McAllen, TX
Clinical Research Atlanta	Nathan Segall MD, Nathan Segall, Jon Finley, Mildred Stull	Stockbridge, GA
Clinical Trials of Texas	Douglas Scott Denham DO, Thomas Weiss MD, Ayoade Avworo DNP, Parke Hedges MD	San Antonio, TX
Coastal Carolina Research Center	Cynthia Becher Strout MD, Rica Santiago, Yvonne Davis, Patty Howenstine, Alison Bondell	Mount Pleasant, SC
Cornell Clinical Trials Unit - Weill Cornell Uptown & Weill Cornell Chelsea, NIAID UM1AI068619, NCAT UL1TR002384	Kristin Marks MS MD, Grant Ellsworth, MS, MD, Tina Wang, MD, Timothy Wilkin, MD, MPH, Mary Vogler, MD, Carrie Johnston, MD, MS	New York, NY
Covid19 Prevention Network (CoVPN, NIAID-NIH)	Michele P Andrasik, Jessica G Andriesen, Gail Broder, Lawrence Corey, Niles Eaton, Kathleen M Neuzil, Huub G Gelderblom, James G Kublin, Rachael McClennen, Nelson Michael, Merlin Robb, Carrie Sopher	Seattle, WA
DM Clinical Research	Vicki E. Miller MD, MPH, Fredric Santiago MD, Blanca Gomez FNP-C, Insiya Valika PA-C, Amy Starr FNP-C	Tomball, TX
Emory University – Ponce de Leon Clinical Research Site, NIAID 3UM1AI068614-14S1	Colleen Kelley MD MPH, Valeria D Cantos MD, Sheetal Kandiah MD MPH, Carlos del Rio MD	Atlanta, GA
Emory University – Hope Clinic, NIAID 1UM1AI148576-01	Nadine Rouphael MD, Paulina Rebolledo, Srilatha Edupuganti, Daniel Sans Graciaa	Decatur, GA
Emory University School of Medicine, NIAID 1UM1AI148576-01	Evan J Anderson MD, Andres Camacho-Gonzalez MD, Satoshi Kamidani MD, Christiana A Rostad MD, Meghan Teherani MD	Atlanta, GA
George Washington University, NIAID UM1AI068619	David Joseph Diemert MD, Elissa Malkin, Marc Siegel, Afsoon Roberts, Gary Simon	Washington, DC
Hackensack University Medical Center	Bindu Balani MD, Carolene Stephenson, Steven Sperber, Cristina Cicogna	Hackensack, NJ
Henry Ford Health System	Marcus J. Zervos MD, Paul Kilgore MD, MPH, Mayur Ramesh MD, Erica Herc MD, Kate Zenlea MPH	Detroit, MI
Hope Research Institute	Abram Burgher MD, Ann Marie Milliken	Phoenix, AZ
Hope Research Institute	Joseph D. Davis MD, Brendan Levy, Sandra Kelman	Chandler, AZ
Hope Research Institute	Matthew W. Doust MD, Denise Sample, Sandra Erickson	Phoenix, AZ
J. Lewis Research	Shane Glade Christensen MD, Christopher Matich, James Longe, John Witbeck	Salt Lake City, UT
J. Lewis Research	James Todd Peterson MD, Alexander Clark, Gerald Kelty, Issac Pena-Renteria	Salt Lake City, UT

Affiliation/Funding*	Study Group	Location
Jacksonville Center for Clinical Research	Michael J. Koren MD, Darlene Bartilucci MD, Jeffery Jacqmein MD, Alpa Patel MD, Carolyn Tran MD	Jacksonville, FL
Javara	Christina Kennelly MD, Robert Brownlee, Jacob Coleman, Hala Webster	Charlotte, NC
Johnson County Clin-Trials	Carlos A. Fierro MD, Natalia Leistner, Amy Thompson, Celia Gonzalez	Lenexa, KS
Kaiser Permanente Washington Health Research Institute, NIAID 1UM1AI148373-01	Lisa A Jackson MD MPH, Janice Suyehira MD	Seattle, WA
Laguna Clinical Research Associates	Milton Haber MD, Maria M. Regalado MD, Veronica Procasky RN JD, Alisha Lutat	Laredo, TX
Lynn Health Science Institute	Carl P. Griffin MD, Raymond Cornelison, William Schnitz, Shanda Gower	Oklahoma City, OK
Lynn Institute of the Rockies	Ripley R. Hollister MD, Jeremy Brown DO, Melody Ronk PA-C	Colorado Springs, CO
M3 Wake Research	Wayne Lee Harper MD, Lisa Cohen DO, Lynn Eckert PA-C, Matthew Hong MD	Raleigh, NC
MediSync Clinical Research Hattiesburg Clinic	Rambod Rouhbakhsh MD, MBA, Elizabeth Danford MD, John Johnson MD, Richard Calderone MD	Petal, MS
Meridian Clinical Research	Shishir Kumar Khetan MD, Oyebisi Olanrewaju AC-CRNP, Nan Zhai NP-C, Kimberly Nieves AC-CRNP, Allison O'Brien AC-CRNP	Rockville, MD
Meridian Clinical Research	Paul Simon Bradley MD, Amanda Lilienthal MSN NP-C, Jim Callis PA-C	Savannah, GA
Meridian Clinical Research	Adam Benson Brosz MD, Andrea Clement PA, Whitney West APRN, Luke Friesen PA, Paul Cramer APRN	Grand Island, NE
Meridian Clinical Research	Frank Steven Eder MD, Ryan Little FNP, Victoria Engler FNP, John Tarbox FNP, Heather Rattenbury-Shaw DO	Binghamton, NY
Meridian Clinical Research	David Jon Ensz MD, Tavane Harrison, Allie Oplinger	Dakota Dunes, SD
Meridian Clinical Research	Brandon James Essink MD, Jay Meyer MD, Frederick Raiser, III MD, Kimberly Mueller APRN, Roni Gray PA	Omaha, NE
Meridian Clinical Research	Keith William Vrbicky MD, Charles Harper MD, Chelsie Nutsch MD, Wendell Lewis III MD, Cathy Laflan MD	Norfolk, NE
Meridian Clinical Research	Jordan L. Whatley MD, Nicole Harrell MD, Amie Shannon MD, Crystal Rowell APRN, FNP-C, Christopher Dedon APRN, FNP-C	Baton Rouge, LA
NIH	Mamodikoe Makhene MD MPH	Bethesda, MD
New Horizons Clinical Research	Gregory Mark Gottschlich MD, Kate Harden PA-C, Melissa Gottschlich PA-C, Mary Smith MSN, FNP-C, Richard Powell MD	Cincinnati, OH
Optimal Research	Murray A. Kimmel DO, Simmy Pinto MD	Melbourne, FL
Optimal Research	Timothy P. Vachris MD, Mark Hutchens MD, Stephen Daniels DO, Margaret Wells MD	Austin, TX
Optimal Research	Mimi Van Der Leden MD, PhD, Peta Gay Jackson Booth MD	Rockville, MD
Palm Beach Research Center	Mira Baron MD, Pamela Kane DO, Shannen Seversen PA-C, Mara Kryvicky PA-C, Julia Lord PA-C	West Palm Beach, FL
Paradigm Clinical Research Center	Jamshid Saleh MD, Matthew Miles, Rafael Lupercio	Redding, CA
Quality of Life Medical & Research Centers	John W. McGettigan Jr. MD, Walter Patton MD, Riemke Brakema MD, Karin Choquette MSN, ABNP-C, Jonlyn McGettigan MSN, RN	Tucson, AZ
Rancho Paseo Medical Group	Judith L. Kirstein MD, Marcia Bernard NP	Banning, CA
Rapid Medical Research	Mary Beth Manning MD, Joan Rothenberg MD, Toby Briskin MD, Denise Roadman PAC, Sharita Tedder-Edwards FNP	Cleveland, OH
Research Centers of America	Howard I. Schwartz MD, Surisday Mederos, Barbara Corral, Jennifer Schwartz, Nelia Sanchez-Crespo	Hollywood, FL
Rutgers New Jersey Medical School, NIAID UM1AI068619	Shobha Swaminathan MD, Amesika Nyaku MD MS, Tilly Varughese MD, Michelle DallaPiazza MD	Newark, NJ
Saint Louis University, NIAID 1UM1AI148685-01	Sharon E Frey MD, Irene Graham MD, Getahun Abate MD PhD MSc, Daniel Hoft MD PhD	St. Louis, MO
St. Vincent's Health System	Leland N. Allen III MD, Leslie Anne Edwards MSN, CRNP, William Simpson Davis Jr., MS PA-C, Jessica Maria Mena, PA	Birmingham, AL
Suncoast Research Group	Mark E. Kutner MD, Jorge Caso MD, CPI, Maria Hernandez Moran APRN, Marianela Carvajal APRN, Janet Mendez APRN	Miami, FL
Sundance Clinical Research	Larkin T. Wadsworth III MD, Horacio Marafioti, Lyly Dang, Jennifer Berry, Lauren Clement	St. Louis, MO
Synexus Clinical Research	Michael Ryan Adams MD, Leslie Iverson PA	Murray, UT
Synexus Clinical Research	Joseph Lee Newberg MD, Laura Pearlman MS, MD, MBA	Chicago, IL
Synexus Clinical Research	Paul Joseph Nugent DO, Leonard Singer	Cincinnati, OH

Affiliation/Funding*	Study Group	Location
Synexus Clinical Research	Michele Diane Reynolds MD, Jennifer Bashour MD, Robert Schmidt MD	Dallas, TX
Synexus Clinical Research	Neil Parmanand Sheth MD, Kenneth Steil DO	Glendale, AZ
Synexus Clinical Research	Ramy Joseph Toma MD, William Kirby MD, Pink Folmar MD, Samantha Williams NP	Birmingham, AL
Synexus Clinical Research	Judith White MD, Robert Meyer MD, Sejal Patel MD, Prity Patel APRN	Orlando, FL
Tekton Research	Paul Pickrell MD, Stefanie Mott FNP-C, Carol Ann Linebarger MD, Hussain Malbari MD, David Pampe MD	Austin, TX
Texas Center for Drug Development	Veronica G. Fragoso MD, Lisa Holloway MD, Cecilia McKeown-Bragas MD, Teresa Becker MD, Vicki Miller MD	Houston, TX
Trial Management Associates	Barton G. Williams MD, William H. Jones MD	Wilmington, NC
VA Greater Los Angeles Healthcare System	Michael Lewis MD, Elham Ghadishah, Joseph Yusin, Mai Pham	Los Angeles, CA
University of California Los Angeles, NIAID UM1AI068619	Jesse L Clark MD, Steven Shoptaw PhD, Michele Vertucci PA, NP, Will Hernandez NP	Los Angeles, CA
University of California San Diego, NIAID UM1AI068636	Stephen A. Spector MD, Amaran Moodley MD, Jill Blumenthal MD, Lisa Stangl NP, Karen Deutsch NP	La Jolla, CA
University of Chicago	Kathleen M. Mullane DO PharmD, David Pitrak MD, Cheryl Nuss FNP, Judy Pi PharmD	Chicago, IL
University of Cincinnati, NIAID UN1AI068619	Carl Fichtenbaum MD, Margaret Powers-Fletcher PhD, Michelle Saemann RN, Sharon Kohrs RN	Cincinnati, OH
University of Colorado Denver, Anschutz Medical Campus, NIAID UM1AI068636	Thomas B. Campbell MD, Andrew Lauria, Jose Castillo Mancilla, Hillary Dunlevy	Aurora, CO
University of Illinois at Chicago – Project WISH, NIAID UM1AI068619	Richard M Novak MD, Andrea Wendrow, Scott Borgetti, Ben Ladner	Chicago, IL
University of Maryland School of Medicine, NIAID 1UM1AI148689-01	Karen L Kotloff MD, Matthew Laurens, Milagritos Tapia, Lisa Chrisley, Cheryl Young	Baltimore, MD
University of Miami, NIAID 3UM1AI068614-14S1	Susanne Doblecki-Lewis MD, Maria Luisa Alcaide, Jose Gonzales-Zamora, Stephen Morris	Miami, FL
University of North Carolina at Chapel Hill, NIAID UM1AI068619	Cynthia Gay MD MPH, David Wohl MD, Joseph Eron, Jr. MD	Chapel Hill, NC
University of Pennsylvania, NIAID 3UM1AI068614-14S1	Ian Frank MD, Debora Dunbar, David Metzger, Florence Momplaisir	Philadelphia, PA
University of Pittsburgh Medical Center, NIAID 1UM1AI148452-01	Judith Martin MD, Alejandro Hoberman MD, Timothy Shope MD MPH, Gysella Muniz MD	Pittsburgh, PA
University of Texas Medical Branch, NIAID 1UM1AI148575-01	Richard Rupp MD, Amber Stanford PA-C, Megan Berman MD, Laura Porterfield MD	Galveston, TX
VA Greater Los Angeles Healthcare System	Michael Lewis MD, Elham Ghadishah, Joseph Yusin, Mai Pham	Los Angeles, CA
Vanderbilt University Medical Center, NIAID 1UM1AI148452-01	Clarence Buddy Creech II MD, Shannon Walker MD, Stephanie Rolsma MD PhD, Robert Samuels, Isaac Thomsen MD	Nashville, TN
Vanderbilt University Medical Center, NIAID 3UM1AI068614-14S1	Spyros Andrews Kalams MD, Greg Wilson MD	Nashville, TN
Velocity Clinical Research	Gregg H. Lucksinger MD, Kevin Parks MD, Ryan Israelsen MD, Jaleh Ostovar FNP-C, Kary Kelly FNP-C	Medford, OR
Velocity Clinical Research, San Diego	Jeffrey Scott Overcash MD, Hanh Chu, Kia Lee, Karla Zepeda	La Mesa, CA
VitaLink Research	Luis I. De La Cruz MD, Steve Clemons, Elizabeth Everette, Suzanna Studdard	Greenville, SC
VitaLink Research	Gowdhami Mohan MD, Stefanie Tyson, Alyssa-Kay Peay, Danyel Johnson	Anderson, SC
VitaLink Research-Spartanburg	Gregory J. Feldman MD, May-Yin Suen, Jacqueline Muenzner, Joseph Boscia, Farhan Siddiqui	Spartanburg, SC
Wake Forest University Health Sciences	John Sanders MD,PhD, James Peacock MD, Julio Nasim MD	Winston Salem, NC
WR-Clinical Research Center of Nevada	Michael L. Levin MD, Julie Hussey MSN APRN FNP-C, Marcy Kulic MD	Las Vegas, NV
WR-ClinSearch	Mark Montgomery McKenzie MD, Teresa Deese, Erica Osmundsen, Christy Sweet	Chattanooga, TN
WR-Global Medical Research	Valentine Mbepson Ebuh MD MA MSc, Elwaleed Elnagar MD, Georgette Ebuh DNP APRN FNP-C, Genevieve Iwuala FNP	Dallas, TX
WR-Medical Center for Clinical Research	Laurie J. Han-Conrad MD, Todd Simmons MD, Denis Tarakjian MD	San Diego, CA

*Funding of institutions by the National Institute of Allergy and Infectious Diseases (NIAID) and/or research support by the National Center for Advancing Translational Science (NCATS) as indicated. All other institutions were funded by Office of the Assistant Secretary for Preparedness and Response, Biomedical Advanced Research and Development Authority. The content of this publication is solely the responsibility of the authors and does not necessarily represent the official views of the funding sources.

CoVPN/COVE Team (cont'd): COVE Trial Investigators and Study Teams

Principal Investigator	Study Team	Institution	Location
Atoya Adams, MD, MBA	Miriah Campbell, Eric Miller, Daisy Langarica, Alia Bober, Diana Giraldo	AB Clinical Trials	Las Vegas, NV
Michael Ryan Adams, MD	Leslie Iverson, Andryelle Toledo, Melinda Bullington, Alicia Hanten, Carolyn Taylor, Shannon Wright, Chase Carnahan, Rachel Law, Natalie Smith, Julie Taylor, Jared-Robert Blake, Stefanie Vasconez, Courtney Jensen	Synexus Clinical Research	Murray, UT
Leland N. Allen III, MD	Leslie Anne Edwards, William Simpson Davis, Jr., Ronald Meza, Jordan Stauffer, John Farringer, Faith Holmes, Rhonda Buzbee, Cristina Velez, Huse Lisa, Lisa Huse, Camelia Speegle, Gregory Prestage, Mary Perez, Jessica Space, Matthew Todd, Jessica McDowell, Marha Bunnell-Pollak, Jackie Ziegler, Jasmine Ali, Dumitru Sirbu, Kellie Williams, Logan Sawyer, Richelle Chambliss, Samantha Blackmon, Stephanie Brennan, Tiffany Gibbs, Alexandria Anderson, Caitlin Roll, Candace Robinson, Zachary McCoy, Jessica Bartlett, Kimberly Cornelison, Chris Bovell, Vincent Baglini, Christy Greenhalgh, Jessica Maria Mena, David House, Matt Honold, Esteban Zurita	St. Vincent's Health System	Birmingham, AL
Evan J. Anderson, MD	Kathleen Stephens, Francine Dyer, Maya Stagg, Aaliyah Carron, Austin Lu, Julia Barton, Sy Tran, Leisa Bower, Esther Park, Jianguo Xu, Rebecca Gonzalez, Vy Ngo, Mike Shepard, Lezly Roxxette Zepeda, Karen Sytsma, Sandra Rojas-Honan, Felicia Glover, Susan Rogers, Theda Gibson, Christina A. Rostad, Andres Camacho-Gonzalez, Teresa Ball, Satoshi Kamidani, Mehgan Farah Teherani, Vikash Patel, Etza Peters, Peggy Kettle, Lisa Macoy, Cindy Lubbers, Amber Samuel, Laila Hussaini, Kathryn Zaks, Caroline Ciric, Meg Taylor, Oliver Smith, Amy Muchinsky, Sydney Biccum, Laura Clegg, Dean Kleinhenz, Angelle Ijeoma, Hannah Huston	Emory University School of Medicine	Atlanta, GA
Lindsey Baden, MD	Xhoi Mitre, Jon Gothing, Bruce Bausk, Jessica Cauley, Natalie Izaguirre, Lewis Novack, Michael Seaman, Katherine Yanosick, Henry Rutherford, Junghyun Kim, Dominique Betterbed, Kathleen Garvey, Lauren Clore, Alexander Mills, Deepesh Duwadi, Alessandra Setaro, Kyl Bowman, Kevin McManus, Sidali Beriane, Fadi Ghantous, Christy Lavine, Jasper Ophel, Joseph Sapiente, Jessica Dorning, Tessa Speidel, Lauren Garneau, Robert Dannemiller, Kirquenique Rolle, Mulika Chhorn, Bailey McCarthy, Hana Flaxman, Milenko Tanasijevic, Cameron Nutt, Javier Barria, Andre Avila-Paz, Buteau Malhaika, Tong Alexandra, Tenaizus Woods, Bethany Evans, Hannah Jin, LaKeisha Gandy, Stephanie St. Pierre, Carolyn Darcy, Michael Corrado, James Maguire, Adetoun Okenla, Tamara Roldon Sevilla, David Kubiak, Cassandre Titus, Movita Harrigan, Maria Alvarado, Rose Theodat, Amy Sherman, Laura Platt, Kirsten Goodman, Laura Nicholson, Wilfredo Matias, Emily Koleske, Ruth Rodriguez, Nicole Taikeff, Jun Bai Park Chang, Julia Klopfer, Phoebe Cunningham, Elizabeth Sampson, Karen Magsipoc, Maureen Macgowan, Lauren Donahue, Haley Schram, Noah Abasciano, Megan Powell, Janet Morgan, Yazed Alsowaida, Olivia Riccardi, Neha Limaye, Virginia Loudermilk, Austin Kim, Kevin Zinchuk, Caitlin Grant, Charles Kelly, David Mellace, Jamie Myers, Erika Gribb, Jose Licona, Monica Feeley, Stephen R Walsh, Jennifer A Johnson, Ann Woolley, Alexis Liakos, Jane Kleinjan, Jon Gothing, Nicolas Issa, Michael Desjardins, Raphael Dolin, Alka Patel, Opeyemi Talabi, Christin Price, Paulette Chandler, Elizabeth W Karlson, Allison P Moriarty	Brigham and Women's Hospital	Boston, MA
Bindu Balani, MD	Smith Kerowyn, Sergio Garcia, Charo Valdez, Shelly Chin, Caitlin DiBello, Silvia Lara, Chika Ekweghariri, Abena Roberts, Abimbola Coker, Marie-Therese Estanbouli, Greg Eskinazi, Michael Tortoriello, Jay Elkareh, Meral Karakoc, Olga Spathis, Patrice Hassoun, Carolene Stephenson, Steven Sperber, Kaur Harveen, Cristina Cicogna, Ciaran Mannion	Hackensack University Medical Center	Hackensack, NJ
Mira Baron, MD	Pamela Kane, Maria Bermudez, Shannen Seversen, Mara Kryvicky, Julia Lord, Terri Barr, Daisy Acevedo, Elena Acosta, Delta Anderson, Alexandra Arango, Anne Bauer, Joshua Egbehor, Tim Flanary, Audrey Haber, Carol Henao, Patti Isaacson, Peter Jacob, Sakaiya Jackson, Karen Kodes, Ludovic La-Branche, Kimarie Lee-Russell, Carol Liso, Cristina Liso, Stephanie Morse, Michelle Navarrette, Christy Norcross, Nora Norcross, Annette Pitts, Mary Sergalis, David Scott, Tytiana Spearman, Danielle Theodore, Brian Thomas, Jennifer Torres	Palm Beach Research Center	West Palm Beach, FL
Judith Borger, DO	Jennifer Angell, Nicole Austin, Deanna Benz, Lucian Cappoli, Nicole Davis, Lynn Eckert, Kathryn Hostetter, Stephanie Keating, Jeanette Mangual-Coughlin, Avia McClain-Stocker, Ifeanyi Momodu, Cheryl Norris, Brennan Opanasenko, Stacey Saldua, Nafisa Saleem, Amy Sheets, Ryan Starr, Scott Syndergaard, Jennifer Thomas, Michelle Wallace, Jeffery Pemberton, Mitchell Arildsen, Dan Tomita	Carolina Institute for Clinical Research	Fayetteville, NC
Paul Simon Bradley, MD	Taja Adams, Stephanie Ailey, Kira Bell, Shanice Bennett, Vincent Bernades, Jim Callis, Bounphone Chanthavong. Taryn Collett, Anne Crouch, Shannon Davis, Morgan Deal, Mimi Duncan, Brandon Essink, Laura Falcone, Debra Gabrielson, Brooke Halpern, Anyfa Hanna, Cassie Heisey, Dawn Kalloniatis, Andrew Kimball, Jeanette Lee, Amanda Lilienthal, Ginny McNew, Crystal Neely, Kay Lynn Olmsted, Nicole Osborn, Chevon Roberts, Pechoka Sanders, Cynthia Seedorf, Kathryn Stoddard, Jonathan Whelan, Stella Yoon	Meridian Clinical Research	Savannah, GA

Principal Investigator	Study Team	Institution	Location
Adam Benson Brosz, MD	Rhonda Richter, Debra Gabrielson, Kayla Flege, Ashley Bell, Karen Jo Johnson, Paul Cramer, Jessica Stanton, Andrea Clement, Whitney West, Laura Falcone, Amanda Friesz, Kathy Osborne, Summer Tophoj, Kimber Breeden, Susan Newman, Douglas Herbek, Lindsey Mettenbrink, Luke Friesen, Alison Pierce	Meridian Clinical Research	Grand Island, NE
Abram Burgher, MD	Stephanie Catanzaro, Shauna Harrell, Magen Hess, Nate Alderson, Bettie D'Nise Corcoran, Norma Frederick, Adrian Alejo, Brian DeCraene, Karen Wakefield, Scarlett Hammett, Susan DeCraene, Ann Marie Milliken, Neil Pearson, Donald Terral Harper	Hope Research Institute	Phoenix, AZ
Thomas B. Campbell, MD	Andrew Lauria, Jenelynn Kimble, Steven Johnson, Matin Krsak, Andrew Monte, Patrisha Adkins, Michelle Barron, Suzanne Fiorillo, Amy Harrison, Anderson Victoria, Nga Le, Sara Berech, Jose Castillo-Mancilla, Kristine Erlandson, Laurel Ware, Josie Marshall, Stephen Bartlett, Hillary Dunlevy	University of Colorado Denver, Anschutz Medical Campus	Aurora, CO
Shane Glade Christensen, MD	Christopher Mickelson, Jessica Shaw, Emily Raming, Amy Nelson, Gabrielle Lewis, Jenessa Folsom, Mikaela Jones, Dylan Owen, Rachel Pugmire, Jennifer Bradley, Annjanette Kemp, Krista Marti, Allyson Christensen, Madison Ellis, Holly Anderson, Emily Bloomquist, Ross Brunetti, Thomas Conner. Jr., Gina Cox, Diana Grazulis, Wesley Lewis, James Longe, Christopher Matich, Bryan Nelson, Sarah Scott, John Witbeck, Stephen Wood	J. Lewis Research	Salt Lake City, UT
Laurence Chu, MD	Jennifer Bacchi, Maria Barrientes, Lamar Box, Christian Casas, R. Michelle Chouteau, Katherine Davis, Tambra Dora, Cindy Duran, Pamela Fidler, Ruth Fitch, Brooke Harris, Isaiah Knight, Jennifer Leyva, Michelle Listz, Jennifer Montes, Javier Perez, Jessica Ruff, Dean Skiles, Sean Turnbow, Francesca Vigil, Breana Wade, Kelly Weber	Benchmark Research	Austin, TX
Jesse L. Clark, MD	Sandy MacNicoll, Somaieh Talebi, Timothy Hall, Steven Shoptaw, Emery Chang, Michael Li, David Goodman, Paul Adamson, Oladunni Adeyiga, Inez Bentancourt, Susan Reed, Christopher Blades, Jasmin Tavares, Demetria Villanueva, Simone Riley, Jonathan Veloz, Schuyler Thomas, Will Hernandez, Jennifer Baughman, Mitchell Stern, Michele Vertucci	University of California, Los Angeles	Los Angeles, CA
Michael J. Cotugno, MD	Kyra Lawson, Kim Harper, Edwin Adamson, George H. Bauer Jr., Julie Bilich, Brenda Lawson, Brandon Illickal, Lois Eaglin, Heather Salisbury, Jeff Segner	Benchmark Research	Metairie, LA
Clarence Buddy Creech II, MD	Shanda Phillips, Naomi Kown, Katherine Sokolow, Wendy Winn, Katherine Wright, Shannon Walker, Stephanie Rolsma, Anna Gallion, April Hanlotxomphou, Deborah Myers, Robert Adkisson, Natalia Jimenez, Cindy Trimmer, Roberta Winfrey, Matthew Donio, John Oleis, Donna Torr, Shelly McGehee, Robert Samuels, Sandra Yoder, Eric Brady, Isaac Thomsen, Madeleine Guy, Emma Alexander, Lana Howard, Krisha Alexander, Shane Moore, Tacora Wright, Tara Evans, Ursula Powell, Jenna Caserta, Valerie Mitchell, Meryk Moore, Melissa Lehman, Diane Anders, Constance Dotye, Crystal Rice, Lamar Bowman, Sherri Hails, Monique Bennett, Nicki Soper, Leigh Howard	Vanderbilt University Medical Center	Nashville, TN
Joseph D. Davis, MD	Sandra Kelman, Sandra Braden, Sabrina Bolland, Mia Munoz, Jose Barocio, Brendan Levy, Dhwani Shah, Neil Pearson, Stephanie Catanzaro, Nathan Alderson, Susan DeCraene, Maureen Godfrey, Skyla Clark	Hope Research Institute	Chandler, AZ
Luis I. De La Cruz, MD	Amy Ford, Taylor Wilson, Cindy Smith, Austin Lambert, Erin Zeiler, Kaelyn Rowland, Marlee Smith, Suzanna Studdard, Zandra Hamilton, Meredith Benfield, Sara Poff, David Godwin, Elizabeth Everette, Steven Clemons, Kayla Peay, Stephanie Gilreath	VitaLink Research	Greenville, SC
Douglas Scott Denham, DO	Thomas Weiss, Parke Hedges, Ayoade Avworo, Kay Scroggins, Leisel Koerber, Antonio Gutierrez, Nathan Cortez, Andrea Gomez, Darlington Akahara, Michelle Smith, Kristy Trevino, Beatriz Herrera, Shaiane Dickerson, Kerry de Jesus, Matthew Korte, Cynthia Ramos, Reanna Martinez, Erica Leal, Shakera Flores, Paul Esparza, Brian Hemming, Melinda Axton, D'Andre White, Terri Perez, Carolina Coronado, Rebecca Many, Clayton Stone, Kimberly Evans, Anshumaan Maharaj, Stephen Brick, Steffanie Barrera, Staci Poettgen, Dawn Killian, Gerardo Pena, Karol Perez, Victoria Hernandez, Kevin Martinez, Amy Griffith, Nolan Payton, Quincey Hogue, Jamie Padilla, Emily Mendez, Lily Hays, Maristelle Co, Nicholas Trinidad, Ismael Rodriguez, Amy Lewis, Cindi Nellis, Lele Simmons, Marissa Johnson	Clinical Trials of Texas	San Antonio, TX
David Joseph Diemert, MD	Linda Witkin, Aimee Desrosiers, DeEnna Wedding, Bertran Walton, LaKeisha Queen, Ryan Mouton, Caroline Thoreson, Manya Magnus, Jennifer Wald, Erika Faust, Nicholas Heredia, Robbie Kattappuram, Hira Qadir, Chelsea Ware, Hannah Yellin, Kegan Dasher, Daniel Mullen, Jeanne Jordan, Taylor Ladson, Madison Lintner, Kaitlyn Macnair, Bitana Saintilma, Kelly Thomas, Samantha Walker, Neha Rampally, Madhu Balachandran, Elissa Malkin, David Parenti, Hana Akselrod, Marc Siegel, Gary Simon, Afsoon Roberts, Aileen Chang	George Washington University	Washington, DC

Principal Investigator	Study Team	Institution	Location
Susanne Doblecki-Lewis, MD	Maria Luisa Alcaide, Jose Gonzalez-Zamora, Stephen Morris, Yimy Puerto, Annie Salvarrey, Claudia Balgas, Claudia Santos, Katherine King, Brahian Steven Erazo, Mayra Fernandez, Leopoldo Cordova-Garcia, Elisa Corzo-Sanchez, Edgar Fernandez, Loreta Padron, Stefani Ann Butts, Kenia Moreno, Juan Casuso, Maria de Pilar Valanzasca, Thomas Tanner, Marilyn Fernandez, Mary Aloise, Inza Patton, Vivian Pastrana, Sendy Puerto, Irma Barreto Ojeda, Junlin Long, Barbara Huang, Gilianne Narcisse, Vanessa Perez	University of Miami	Miami, FL
Matthew W. Doust, MD	Denise Sample, Sandra Erickson, Nate Alderson, Adrian Alejo, Stephanie Catanzaro, Susan DeCraene, Cassie Enricco, Sandra Erickson, Alex Guereque, Shauna Harrell, Shana Harshell, Stephanie Junker, Stephanie Laufenberg, Madison Mikulak, Makayla Morra, Nicole Olson, Neil Pearson, Jasmin Redden, Monique Romo, Denise Sample, Dhwani Shah, Sahara Vega, Emma Kar	Hope Research Institute	Phoenix, AZ
Valentine Mbepson Ebuh, MD	Elwaleed Elnagar, Georgette Ebuh, Genevieve Iwuala, Catina Adams, Marissa Cervenka, Ezgar Del Real, Shraddha Dubal, Elwaleed Elnagar, Jenifer Fiatte, Kathy Harrell, Genevieve Iwuala, Vicki Martinez, Robert Miranda, Brennan Opanasenko, Destiny Robinson, Liz Ruiz, Amy Sheets, Shoniece Wallace	WR-Global Medical Research	Dallas, TX
Frank Steven Eder, MD	Ryan Little, Victoria Engler, John Tarbox, Heather Rattenbury-Shaw, Deborah Hubish, Jessie Taylor, Debra Gabrielson, Jessica Fellows, Jennifer Molstead, Kathe Olmstead, Ashley Conover, Tammy Kohn, Chelsea Briar, Corrine Young, Collen McVannan, Kelli Quick, Shaylynne Hubanks, Kimber Breeden, Ann Marie Sampson, Traci Hull, Tarin Gordon, Susan Owen, Kate Macarak, Tonya Rackett, Jacob Blattstein, Partidge Jane Aton, Nicole Croft, Carolyn Grausgruber, Rebecca Miller, Ryan Little, Victoria Engler, John Tarbox, Heather Rattenbury-Shaw, Nathan Kimball, Courtney Heisey, Ginny McNew, Abigail Wine, Cindi VanKuren, Jared Frick, Tammy Dennis, Andrew Kimball	Meridian Clinical Research	Binghamton, NY
Hana M. El Sahly, MD	Jennifer A. Whitaker, C. Mary Healy, Christine Akamine, Wendy A Keitel, Robert L Atmar, Annette Nagel, Sandra Francisco, Thea Marie Cordero, Janet Brown, Jennifer Christensen, Caroline Doughty-Skierski, Connie Rangel, Carrie Kibler, Coni Cheesman, Lisreina Toro, Chanei Henry, Chianti Wade Bowers, Pedro Piedra, Kathy Bosworth, Kayla Burrell, Jesus Banay, Tykel Eddy, Trent Davis, Shetel Anassi, Yvette Rugeley, Olga Rybina-Willis	Baylor College of Medicine	Houston, TX
David Jon Ensz, MD	Pamela Allen, Taylor Bergh, Kimber Breeden, Avery Dunn, Brandon Essink, Debra Gabrielson, Rylea Gulick, Tavane Harrison, Courtney Heisey, Andrew Kimball, Shelby Klaschen, Jessica Knight, Makayla Langston, Meagan Miller, Allie Oplinger, Heather Persinger, Alison Pierce, Kathryn Stoddard, Kayla Sturgeon, Jamie Thompson, Melissa Wiseman	Meridian Clinical Research	Dakota Dunes, SD
Brandon James Essink, MD	Jay Meyer, Frederick Raiser, Kimberly Mueller, Roni Gray, Riley Brockman, Tabitha Campbell, Carrie Essink, Laura Falcone, Roni Gray, Linda Layton, Jay Meyer, Kimberly Mueller, Tiffany Nemecek, Frederick "Fritz" Raiser, III, Jessica Satorie, Chelsea Steinmetz, Nicole Osborn, Cassie Heisey, Maria Nguyen	Meridian Clinical Research	Omaha, NE
Gregory J. Feldman, MD	May-Yin Suen, Brittany Cooksey, Madison Fowler, Sarah Chynoweth, Gary Clemons, Laura Jolly, Charlie Jordan, Heather Allison, Steve Clemons, Amber Brittany Belcher, Allison Kelly, Marsha Gossett, Wendy Taylor, Amy Witt, Kendal Nelson, Jeffrey Witt, Jacqueline Muenzner, Elizabeth Everette, Supinder Channa, Allison Ayers, Joseph Boscia, Farhan Siddiqui	VitaLink Research-Spartanburg	Spartanburg, SC
Carl J. Fichtenbaum, MD	Maggie Powers-Fletcher, Michelle Saemann, Sharon Kohrs, Kimberly Mullins, Lindsay Davis, Moises Huaman, Angela Snyder, Kristin Weghorn, Brenda Miller, Elizabeth Costea, Lisa Schira, Romana Saeed, Helen Shelton, Kathleen Ballman, Laura Browning-Cho, Sherry Donaworth, Chris Goddard, Jeanine Goodin, Elizabeth Niederegger, Lisa Hachey, Tamara Maus, Pam Fletcher, Makayla Bishop, Victoria Straughn, Shaina Horner, Carrie Christofield, Dana Burns, Jason Mayes, Kelly Windholtz, Lisa Proffitt, Faizan Qureshi, Michelle O'Neil, Arustamyan Lisa, Sarah Trentman, Eva Whitehead, Jennifer Baer, Linda Hinds, Jaasiel Chapman, D'Vaughn House, Gary Frazier, Judy Houston, Lisa Altenau, Mary Burns, Dorice Smith, Justin Ragle, Eric Mueller, Cynthia Nypaver, Jaime Robertson, Anissa Moussa, Geronimo Feria Garzon, Sierra Bennett, Marlena Petrie	University of Cincinnati	Cincinnati, OH
Carlos A. Fierro, MD	Mazen Zari, Celia Gonzalez, Natalia Leistner, Mary Easley, Mary Provost, Krista Estrada, Ann Geier, Amy Thompson, Heather Barker, Karol Moore, Kelly Moen, Monica Atwood, Amber Wolf, Brandi Dickerson, Manyvohn Rinehart, Dina Hammine, Angela Eichler, Casey Johnson, Nathan Arthur	Johnson County Clin-Trials	Lenexa, KS
Veronica G. Fragoso, MD	Lisa Holloway, Cecilia McKeown-Bragas, Teresa Becker, Vicki Miller, Leena Mir, Elton Oliveira, Moez Talpur, Enya Rentas-Sherman, Gabriela Maria Becerra, Dewayne Hicks, Robert Krbashyan, Shakira Barr, Ashraf Jafri, Herman Ortiz, Zohair Harianawala, Chandra Tobin, Norma Gonzalez, Saji Perinjelil, Khorshid Amirkhosravi, Tracy Kowalski, Biman Goswami, Waheeda Sureshbabu, Amy Anderson, Berenice Ferrero, Simeen Khan, Chen-Ho Yang, Nazanin Zarinkamar, Scott Ward, Crystal Reese, Miyosha Lewis, Olga Konshina, Lorrian Yates.Joel Cano, Quiana Wilson, Kara	Texas Center for Drug Development, Inc.	Houston, TX

Principal Investigator	Study Team	Institution	Location
	Sikes, Diana Chehab, Joanna Quezan, Maryam Rabbani, Sadaf Batla, Abbyssinia Moges. Diego Carrington, Matthew Joseph, Laura Grissanty, Dean Jang, Dustin McFadden, Misbah Baloch, Elisa Moralez, Abdeali Dalal, Frances Saubon, William Fernandez, Jenny Toress, Blessing Felix, Zain Rizvi		
Ian Frank, MD	Annet Davis, Eileen Donaghy, Nicole Sundo, Juan Ramirez, Laura Schankel, Dana Brown, Katharine Bar, Dana Brown, Christopher Chianese, Gillain Constantino, Dovie Watson, Kathleen Degnan, Helen Koenig, William Short, Petra Alexander, Eileen Mergliano, Jie Ho, Michele Wisniewski, Debora Dunbar, Liani Santini-Lopez, Rosemarie Kappes, Angela Cabassa, Tammy Chen, Berry SotoVega, Deborah Kim, Devon Cliett, Kate Kearns, Jillian Baron, Vivian Leung, Florence Momplaisir, Sarah Wood, Tameka Matthews, David Metzger, Richard Tustin	University of Pennsylvania	Philadelphia, PA
Sharon E. Frey, MD	Irene Graham, Getahun Abate, Daniel Hoft, Heather Douds, Cassandra Zehenny, Joan Siegner, Helay Hassas, Kim Cooper, Shirley Dettlebach, Sabrina DiPiazza, Carol Duane, Linda Eggemeyer-Sharpe, Lauren Foreman, Jerry Hutter, Ryan Kerr, Kate Liefer, Tracy Montauk, Karla Mosby, Janice Tennant, Nicole Purcell, Kiana Wilder, Kathleen Chirco, Sharon Irby-Moore, Kathleen Koehler, Melissa Loyet, Thomas Pacatte, Susan Stewart, Azra Blazevic, Tamara Blevins, Chase Colbert, Christopher Eickhoff, Lainey Mejia Jauregui, Keith Meyer, Krystal Meza, Amanda Nethington, Huan Ning, Brittany Williams, Mei Xia, Yinyi Yu, Stanley Doublin, Mary Pat Eastman, Eric Eggemeyer, Mikayla Frye, Michelle Harris, Aleshia McCoy, Donna Duncan, Gwendolyn Tatum, Nicole Purcell, Kiana Wilder, Tammy Grant, Claudia Castillo Paredes, Rong Hou, Jin Wang, Qian Wang, Sarah George	Saint Louis University	St. Louis, MO
Cynthia Gay, MD	David Wohl, Joseph Eron, Jr., Andrew Thorne, Michelle Floris-Moore, Christopher Hurt, David Wohl, Chidinma Okafor, Janette Goins, Ulrike Adam, Ekundayo Nylander-Thompson, Anna Furlong, XinHong Ao, Kathy Guerrero, Melinda Hart, Kathleen Loeven, Rachael Mossey, Esther Speight, Rachel White, Chloe Twomey, Kristen Gray, Miriam Chicurel-Bayard, Susanne Henderson, Patti Vasquez, April Welch, Camille O'Reilly, Maureen Furlong, Noshima Darden-Tabb, Elizabeth DuBose, Marie Oriol, Dynesha Perry, Maria Stetson, Maria Bullis, Shelby Turner, Ebony Harrington, Michael Herce, Suzanne Blevins, Alexander Bradley, Susan Pedersen, Becky Straub, Sandra Barnhart, Felicia Barriga Munante, Nazneen Howerton, Tevnan Keller, Mandy Tipton, Abigail Riddick, Kristi Kirkland, Maggie Harman, Tania Hossain, Centhla Washington, Erin Hoffman, Carolina Pastrana Medina, William Johnson, Samantha Earnhardt, Amy James Loftis, Catherine Kronk, Yaa Ofori-Marfoh, Julie A Nelson, Nicole Maponga, Lina Rosengren-Hovee, William Zhao, Jennifer Thompson, Sarah Law, Holly Milner, Jonathan Oakes, Rachel Cook, Erin Cardot, Oesa Vinesett, Victoria Rucinski, Joy Wannamaker, Tanailly Giralt Smith, Eliza DuBose, Chidinma Okafor	University of North Carolina at Chapel Hill	Chapel Hill, NC
Richard M. Glover, II, MD	Stacy Slechta, Troy Holdeman, Robyn Hartvickson, Amber Grant, Jennifer Bennett, Lindsey Brewer, Janelle Brown, Kelsey Burden, Melissa Burton, Brianna Burton, Jordan Danby, Sheri Duncan, Amber Grant, Robyn Hartvickson, Lisa Hemmelgarn, Sherry Henning, Jeri King, Riley King, Colton King, April Kitterman, Shannen Lassiter, Cayla Lawless, Janna Martinez, Ragene Moore, Marissa Mueller, Aaron Nguyen, Justin Phillips, Jordan Reheis, Rebecca Ring, Katherine Saengerhausen, Shannon Thomas, Dylan Thomas, Cindy Thome, Denae Villines, Amber Wenzel, Eilleen Wilbert, Avi Woods, Caressa Presley, Brianna Newport, Olivia Allen, Miranda Santiago, Cheryl Sauerwein, Jill Longstaff, Sadie Allen, Candace Heckart	Alliance for Multispecialty Research	Newton, KS
Gregory Mark Gottschlich, MD	Melissa Gottschlich, Steven Anderson, Gregory Mark Gottschlich II, Mary Woeste, Kate Harden, Cindy Young, Michael Pordy, Audrius Ruksenas, Lacy Baird, Kim Krogman, Lori Stanton, Melissa Fuson, Mason Urban, Christine Watson, Richard Powell, Mary Smith, Jacob Sekinger, Diamond Russell, Nicole Lim, Mylene Asmar-Rios, Yusef Museitif, Craig Mitchell, Tarik Whitham, Zachary Rutledge, Troy Porter, Andrea Newlands, Jami Ramsey, Mary Frances Curry, Nishay Holloman, Crystal Barket, Michelle Spear, Shelley Mahan, Taeleigha Greene, Zachary Eardley, Gen Moussa, Mary Ann Gottschlich	New Horizons Clinical Research	Cincinnati, OH
Sinikka L. Green, MD	Julie Hamilton, Alex Fuller, Jeanette Dickhaus, Colleen Jacobson, Triny Cooper, Michelle Jackson, Taylor Evans, Tabitha Judd, Kathryn Alexander, Megan Rosallo, Sikhongi Phungwayo, Robin Dotson, Dana Finley, Michael Vasquez, Cyndi Foster, Gregg Lucksinger, Sarah Smiley, Jayasree Krishnankutty, Ray Coon, Grishma Dhimmer, Melanie Wilkerson, Tatum Shawver, Marcedes Coffman, Devin Teal, Laura Crenshaw	Advanced Clinical Research	Cedar Park, TX
Carl P. Griffin, MD	William Schnitz, Andrea Romero, Kim Hamilton, Raymond Cornelison, Angela Genovese, Shelly Brunson, April Green, Lacey Dietz, Kim Calloway, Chris Hyatt, Destiny Heinzig-Cartwright, Chalimar Rojo, Sharee Wright, Kathi Shaw, Michael Pojezny, Avery Keller, Krystal Hightower, Dalia Tovar, Shanda Gower	Lynn Health Science Institute	Oklahoma City, OK
Milton Haber, MD	Maria Candelario, Martha Bunnell-Pollak, Lauren Wade, Jackie Ziegler, Deena Ramirez, Perla Avalos, Maria Drada, Jasmine Ali, Jessica McDowell, Kehinde Busari, Patricia Church, Ronald Meza, Marco Vela, Esteban Zurita, Chris Connolly, Ruben	Laguna Clinical Research Associates	Laredo, TX

Principal Investigator	Study Team	Institution	Location
	Del Bosque, Alisha Lutat, Chelsea Fleming, Brett Potthoff, Anita Suri, Cynthia Priester, Brenda Hernandez, Veronica Procasky, Eva Cerreta, Matt Honold, Melinda Rodriguez, Maria Regalado, Jordan Stauffer		
Greg Hachigian, MD	Michael Cancilla, Ricardo Castellanos, Angela Cuellar, Yaman Darmarathne, Shaila Faulker, Yana Gordeyeva, Michelle Hisey, Ashley Jungsten, Kristin Kiersey, Pawandeep Nagra, Nav Nagra-Kooner, Jazmin Nauta, Masaru Oshita, Kenneth Quick, Julie Raygoza, Amanny Sadek, Melisa Tinder, Jhoana Torres, Deborah Murray, Kristen Kiersey	Benchmark Research	Sacramento, CA
Laurie J. Han-Conrad, MD	Brandon Baldwin, Lucian Cappoli, Tenisha Garcia, Ella Grach, Brenda Grande, Nicolle Mendez, Natalie Moy, Matthew Musikanth, Karen Mylerberg, Brennan Opanasenko, Mark Pulera, Patti Sanchez-Emery, Mireles Sarah, Todd Simmons, Denis Tarakjian	WR-Medical Center for Clinical Research	San Diego, CA
Wayne Lee Harper, MD	Toni Bland, Lori Bridges, Lucian Cappoli, Lisa Cohen, Leah Corts, Annie Craft, James Earnhardt, Lynn Eckert, Aubrey Farray, Laura Hoer, Matthew Hong, Chris Hoyle, Jenee Jiggetts, Brian Joseph, Bradley Killebrew, Kendra Lisec, Lucie Mangala, David Musante, Adnan Nasir, Amanda Olsen, Brennan Opanasenko, Marci Parks, Marion Peoples, Katherine Schuch, Judith Shand, Sabine Ucik, Douglas Wadeson, Barbara Wheeler	M3 Wake Research	Raleigh, NC
Ripley R. Hollister, MD	Jeremy Brown, Brandy Ball, Jeremy Brown, Valerie Dyster, Dalia Jeronimo, Shelby Pickle, Michael Pojezny, Melody Ronk, Kathi Shaw, Bobbi Shofner, Jami Wagner, Meghan York, Jill York	Lynn Institute of the Rockies	Colorado Springs, CO
Lisa A. Jackson, MD, MPH	Marilyn Nguyen, Maya Dunstan, Barbara Carste, Sarah Friend, Diana McFeters, Lynn Gross, Mohamed Ajenah, Jana Ffitch, Audra Mccoy, David Skatula, Susan Lasicka, Kimberly Brinker, Karen Sherwin, Melissa Scheer, Paula Lins, Roger Calvert, Roxanne Erolin, Stella Lee, Vi Tran, Stephanie Pimienta, Bruce Douglas, Lee Barr, Colin Fields, Erika Kiniry, Joe Choe, Janice Suyehira, Joyce Benoit, Michael Witte, Rebecca Lau	Kaiser Permanente Washington Health Research Institute	Seattle, WA
Spyros Andrews Kalams, MD	Greg Wilson, Kyle Rybczyk, Katie Crumbo, Carly Griffin, Latoya Hannah, Amy Kerrigan, Valerie Mitchell, Jenna Caserta, Mary Downey, Nicole Swindle, Shonda Sumner, Amber Massey, Trudy Sullivan, Rita Smith, Cindy Nochowicz, Eric Olson, Christian Warren, Josh Simmons, Dana King, Gwendolyn Rees, Matt Donio, Jesse Case, Keith Richardson, Jarissa Greenard	Vanderbilt University Medical Center	Nashville, TN
Colleen Kelley, MD, MPH	Valeria D. Cantos, Sheetal Kandiah, Carlos del Rio, Christina Bacher, Hannah Huston, Juliet Brown, Divya Bhamidipati, Nithin Gopalsamy, Brittany Lynn Speigel, Elizabeth (Betsy) Hall, Brandon Spratt, Kiran Dhillon, Caitlin Moran, Michael Chung, Felecia Wright, Marcia Peters, Rondell Jaggers, Vanessa Soliman, Ron Gaston, Christopher Foster, Sarah Wiatrek, Bezuayehu Mandefro, Pamela Weizel, Pamela Lankford-Turner, Anandi Sheth, John Gharbin, Catherine Abrams, Philip Powers, Paulina Rebolledo, Christin Root, Tiraje Lester, Sha Yi, Damien Swearing, Fred Ede, Isaac Perez, Kelly Likos, Meen Dhir, Aastha KC, Gabriela Georgial, Tucker Colvin, Nabeel Yar Khan, Valarie Hunter, D'Jamel Young, Felecia Atkinson	Emory University Emory University – Ponce de Leon Clinical Research Site	Atlanta, GA
Christina Kennelly, MD	Jacob Coleman, Brittany Bundeff, Melissa C. Hennessey, Kenneth Owen, Caroline Wilds Wilds, Jennifer Womack, Susan Martello, Chiedza Hooker, Robert Brownlee, Melissa James, Deborah Wesley-Farrington, Lori Whiteheart, Hala Webster, David Framm, Cortney Fretz, Gwyn Gibson, Susan Donahue, Kelly Woodell, Linda McCarty, Jim Vesely, Scott Chatterton, Andrew Ottesen, Enrico Belgrave, Krishna Shah, James Chester Alexander, Brittain Callahan	Javara	Charlotte, NC
Shishir Kumar Khetan, MD	Taja Adams, Tanya Alexander, Tanya Alexaner, Sydney Barmoy, Jake Bart, Kira Bell, Ira Berger, Jemario Blackwell, Priscilla Buahin, Bounphone Chanthavong, Juliana DeVito, Azure Erskine, Brandon Essink, Laura Falcone, Debra Gabrielson, Beau Garland, Barb Geiger, Tiana Oliver, Courtney Heisey, Sucharita Katikala, Andrew Kimball, Heather Lang, Jeanette Lee, Asefa Mekonnen, Devan Myers, Kimberly Nieves, Allison O'Brien, Oyebisi Olanrewaju, Nicole Osborn, Adunola Oshiyoye, Rahul Patel, Alan Pollack, April Poole, Collin Smith, Kathryn Stoddard, Chao Wang, Sean Whelan, Jonathan Whelan, Graciela Zapata, Nan Zhai	Meridian Clinical Researc	Rockville, MD
Murray A. Kimmel, DO	Alexa Diec, Ann Riley, Bette Denmat, Bram Swarr, Christina Raidl, Dania Billman, Denise Dixon, Donald Dawson, Elaine Crudo, James Crowley, Katrina Carlson, Kaylie Worzick, Laura Worth, Lisbeth Gordon, Marion Oliver, Robert Holt, Simmy Pinto, Taylor Atkinson, Traci Mitchell, Lana Ghomrawi, Norma Rokoff	Optimal Research	Melbourne, FL
Judith L. Kirstein, MD	Jared Bradshaw, Krista Forster, Jeanette Dickhaus, Marcia Bernard, Erica Sanchez, Nikki Abels, Cynthia Kunakom, Vanessa Vandergoot, Jessica Fisher, Carol Remigio, Jourdan Manfred, Frederick Lloyd, Tiffany Williams, Clarisse Baudelaire, Lovette Cherelle, Nolan Mackey, Alan Valenzuela, Theodore Wyman, Alyssa Taber, Karen Myers, Craig Koch	Rancho Paseo Medical Group	Banning, CA
Michael J. Koren, MD	Shannon Trull, Amanda Elwood, Mary Strickland, Ivy Gulliermo, Chistopher Ganzhorn, Sonia Gerardo, Taylor Johnson, Victoria Kaposchansky, Cassie Lawler, Laura Little, Amanda Pratt, Sheldon Warren, Andrea West, Emery Noles, Nathanial	Jacksonville Center for Clinical Research	Jacksonville, FL

Principal Investigator	Study Team	Institution	Location
	Grant, Jillian Agnew, Lori Alexander, Brenda Anderson, Deirdre Arrington, Sara Benner, Lisa Carl, Allison Crain, Nafisa Ishaku, Robert Nix, Sharon Smith, Amber Devries, Sandy Salceiro, Opara Chukwudi, Mikaela Karney-Trull, Ramil Castillo, David Graham, Gail Lowe, Alexander Hill, Carolyn Tran, Jeffry Jacqmein, Darlene Bartilucci, Alpa Patel, Janet Garvey, Mitchell Rothstein, Kenneth Aung-Din, Margaret Gannaway, Arman Mughal, Sandra Fuit, Jolenne Wolfer, Erin Schelhorn, Jacob Wolfer, Madison Martinez, Melissa Parks, Patricia Neal		
Karen L. Kotloff, MD	Matthew Laurens, Milagritos Tapia, Lisa Chrisley, Cheryl Young, Barbara Albert, Robin Barnes, Shernel Barrett, Andrea Berry, Melissa Billington, Shannon Bittner, Colleen Boyce, Faith Pa'Ahana Brown, James Campbell, Regina Carpenter, Jamonie Carter, Ginny Cummings, Brenda Dorsey, Jorge Flores, DeAnna Friedman-Klabanoff, Shirley George, Nancy Greenberg, Hassan Haji, Elizabeth Hammershaimb, Susan Holian, Leslie Howe, Myounghee Lee, Alyson Kwon, Kirsten Lyke, Alma Valle Maldonado, Jennifer Marron, Kaitlin Mason, Monica McArthur, Rosa McBryde, Sherry McCammon, Sandra Molina, Kathleen Neuzil, Daniele Nitkowski , Justin Ortiz, Rekha Rapaka, Mardi Reymann, Toni Robinson, Wanda Somrajit, Mark Travassos	University of Maryland, School of Medicine	Baltimore, MD
Mark E. Kutner, MD	Amanda Colina, Isett Caro, Frances Beltran, Jessie Downs, Jonathan Fernandez, Mariete Renden, Mirnaya Mujica-Alabaci, Susel Figueredo, Yanelis Dominguez, Jaime Blandon, Bryan Ruiz, Leidy Montoya, Edgardo Rodriguez, Jessie Downs, Jason Rothscheld, Janett Acle, Yaime Martinez, Soraya Ricardo, Maria Hernandez Moran, Eloisa Guerra, Heidie Perez, Claudia Rodriguez, Victoria Moreno, Vanessa Hechavarria, Saray Carvajal, Daniel Lopez, Carlos Iviricu, Neiner Enriquez, Paola Garcia, Chris Hoyle, Marianela Carvajal, Janet Mendez, Edisleidy Mesa, Marco Ramirez, Dalila Del Valle, Jennifer Ortega, Yeni Hernandez, Jhobana Vargas, Carmen Amador, Juan Delgado, Maury Santos, Meredith Arguelles, Leyanis Coello, Vanessa Ansorena, Jorge Caso, Stacy Machado, Raydel Valdes, Giann Lightbourn, Dayami Dovales, Alain Chang	Suncoast Research Group	Miami, FL
Mimi Van Der Leden, MD, PhD	Chrishea Harvey, Tricia Oyeyemi, Aicha Moutanni, Stephanie Melton, Peta-Gay Jackson Booth, Jennifer Yoon, Gloria Kim, Atanas Filev, Francis Uwandi, Meyling Lopez, Janice Spreitzer, Courtney Gennes, Xiangfei Cheng, Matthew Van Sickle, Nick Bart, Brianne Okunji, Frank Maloba	Optimal Research	Rockville, MD
Michael L. Levin, MD	Brennan Opanasenko, Yajaira Ramos, Shonda Lester, Rebecca Boucher, Shawn Harrell, Shon Boucher, Patti Sanchez, Nina Scharbach, Alex Sanchez, Shyane Raniello, Wendy Guerra, Krystal Tyner, Kimberly Temple, Ruby Ortiz, Daniel Terreault, Amy Kill, Jade Odynski, Adolfo DeLeon, Debbie Carter, Eduardo Rodriguez, Julia Gass, Sara Esparza, Sierra Dansbee, Tammy Harrison, Marcy Kulic, Lucian Cappoli, Mora KIm, Matthew Fenner, Heather Jimenez, Shraddha Dubal, Julie Hussey	WR-Clinical Research Center of Nevada	Las Vegas, NV
Michael Lewis, MD	Nancy Mohler, Mai Pham, Ron Waldorf, Elham Ghadishah, Samantha Feril, Stella Lee, Dzuyen Nguyen, Ruoxiang Wang, Justine Velandria, Benjamin Dreskin, Joseph Yusin, Lauren Vigil, Sara Wong, Suchi Tiwari, Joseph Pisegna, Sunita Dergalust, Wayman Lee, Krissa Caroff	VA Greater Los Angeles Healthcare System	Los Angeles, CA
Gregg H. Lucksinger, MD	Jaleh Ostovar, Craig Koch, Danuel Hamlin, Kelly Chase, Jeanette Dickhaus, Edward Kerwin, Frederick Forde, Allison Alvord, Dawn Stewart, Dan Hamlin, Kevin Parks, Ryan Israelsen, Kary Kelly, Tiffany Smith, Melissa Myers, Ryan Rackley, Audrey Kuehl, Savannah Peterson, Hannah Hall, Jay Weisbart, Alison Dodenhoff, Emily Kelly	Velocity Clinical Research	Medford, OR
Mary Beth Manning, MD	Carol Salango, Alec Ireland, Lisa Hoagland, Jeanette Dickhaus, Toby Briskin, Joan Rothenberg, Michael Gaston, Sharita Tedder-Edwards, Denise Roadman, Megan Sokolowski, Tina Shickluna, Katherine Bielanski, Samantha Hood, Talia Chandler, Brianna Arman, Melinda DeLong, Naqib Ahmad, Karly Tarase, Jade Svoboda, Lisle Merriman, Melisa Sebera, Emma Landskroner, Amy Maroun, Brooke Glivar, Jennifer Gaston, Sarah Dzigiel, Cassiandra Uminski, Karol Sabol, Devan Patel, Nick Zarbo, Briana Jackson, Brian Sharpe, Nicole, Baitt, Kaitlyn Duffy, Gabrielle Jacobs, Ann Czuprun, Tracee Cash, Diamond Ivey, Kaitlyn Rubell	Rapid Medical Research	Cleveland, OH
Kristen Marks, MD	Grant Ellsworth, Tina Wang, Timothy Wilkin, Mary Vogler, Carrie Johnston, Marshall Glesby, Roy Gulick, Ole Vielemeyer, Rebecca Fry, Todd Stroberg, Caitlin Rhoades, Noah Goss, Shaun Barcavage, Valery Hughes, Jonathan Berardi, Caroline Greene, Sarah Galloway, Caique Mello, Ashley Machado, Mia Crowley, Monique Williams, Katherine Fee, Elizabeth DeJesus, Andrew Yu, Minkyung Lee, Susan Herder, Mary Ann Zweibel, Patrice Weller, Antonio Rivera-Lopez, Edward Kenny, Hetal May, Natella Fridman, Parul Shah, Ruby Lee, Venus Fernandez, Victoria Lesina, Celine Arar, Byron Bullough, Kinge-Ann Marcelin, Brian Mangano, Jessenia Fuentes, Jiamin Li, Genessi Rodriguez, Catherine Jerry, Nadi Islam, Liqun Cai, Wayne Burns, Akinbayo Caulcrick, Andrika Thomas, Barbara Batog, Guoan He, Sara Yoder, Tamara Crowder, Gianna Resso, Sophia Alvarez, Tahera Begum, Elizabeth Connolly, Roxanne Rosario, Paul Kim, Steven Wang, Vasilika Koci	Cornell Clinical Trials Units - Weill Cornell Chelsea and Uptown	New York, NY

Principal Investigator	Study Team	Institution	Location
Judith Martin, MD	Alejandro Hoberman, Timothy Shope, Gysella Muniz, Sonika Bhatnagar, Kumaravel Rajakumar, Anne-Marie Rick, Peri Unligil, Jennifer Nagg, Melissa Andrasko, Mary Ann Sieber, Jennifer Opal, Lalicia Roman, Spenser Kinsey, Michelle Burke, Matthew Lee, Dominic Kramer, Linette Milkovich, Emily Dougherty, Emily Carney, Shannon Mance, Nader Shaikh, Diana Kearney, Jamie Fries, Lisa Vavro, Shayla Goller	UPMC University Center	Pittsburgh, PA
John W. McGettigan, Jr., MD	Walter Patton, Jennifer Schnider, Riemeka Brakema, Heeten Desai, Mikell Brett Karsten, Patricia Jalomo, Cindy Finch Benoy, Karin Choquette, Jonlyn McGettigan, Yvonne De Los Reyes, Melissa Cozzens, Amanda Hermosillo, Cindy Montgomery, Susan Tarwid, Annette Elzy, Tianna Young, Saysamone Banks, Cristina Fernandez, Damaris Atondo, Zoe Sesma, Norma Barrientos, Maggie Tono, Kisha Adams, JoAnn Wilkins, Arianna Bermudez, Carol Sayer, Julie McDowell, Angelina Navarro, Mercedes Sullivan, Crystal Mata, Sheldon Gingrich, Aaliyah Sestiaga, Gia Longo	Quality of Life Medical & Research Centers	Tucson, AZ
Mark Montgomery McKenzie, MD	Tiffany Jewell, Zackery Harmon, Michael Elizabeth, Christy Sweet, Teresa Deese, Catherine Schon, Misti Earwood, Lou Cappoli, Brennan Opanasenko, Lisa Guider, Michelle Forgey, Justian Jarrett, Rachel Scott, Elizabeth Michael, Erica Osmundsen, Andrew Wood, Shelly Brooks, Gisela Heintz, Lilian Nukuna	WR-ClinSearch	Chattanooga, TN
Vicki E. Miller, MD	Sajjad Naqvi, Soofia Masood, Fredric Santiago, Sonia Guerrero, Subhash Koneru, Nirja Shah, Andrea Torres, Ramani Gali, Talha Baig, Heather Leary, Afifah Ayub, Nayab Goher, Patti Tate, Reagen Reed, Muhammad Irfan, Amy Starr, Alefiyah Motiwala, Julia Kenny, Victoria Aguilar, Jessica Arguijo, Insiya Valika, Victoria Aguilar, Jagruti Patel, Anna Pena, Faryal Mahmood, Blanca Gomez, Nancy Torres, Kristyn Latil, Tarori Mark, Laura Djampou, Lindsey Kueng, Marianne Tadros, Mohammad Millwala, Monica Murray, Murtaza Marvi, Shivani Shah, Vanessa Gonzalez, Zohair Harianawala, Zainab Rizvi, Ambily Dileep, Jaquelyn Gonzales, Ragen Powell, Carolina Deandres, Syed Fahad Ali Kazmi, Sandra Natalia Perez, Shannon Amacker, Shiela Varghese	DM Clinical Research	Tomball, TX
Gowdhami Mohan, MD	Rodolfo Barrera, Emma Partin, Kelly White, Ashley Rochester, Charles Thompson, Stefanie Tyson, Ashten Sheriff, Alyssa-Kay Peay, Kayla Corn, Barbara A. Richardson, Kristin Miller, Steven Clemons, Cameron King, Emma Partin, Gary Clemons, Brianna Starr, Danyel Johnson, Taylor Davis, Niki Tyson	VitaLink Research	Anderson, SC
Kathleen M. Mullane, DO, PharmD	David L. Pitrak, Cheryl Nuss, Karen Cornelius, Randee Estes, Amy Luckett, Michelle Moore, Judi Pi, Stephen Schrantz, Jill Stetkevych	University of Chicago	Chicago, IL
Joseph Lee Newberg, MD	Mary Reyes, Nicole Leahy, Victoria Andriulis, Herbert Whinna, Patricia James, Lana Ghomrawi, Carole Kempfer, Miriam Arroyo, Maria Castro, Anna Maddox, Reuben Martinez, Jacquilyn McCormick-Burks, Laura Pearlman, Rosalinda Vazquez, Shaheera Suleiman, Neha Atal, Rosalind Vazquez	Synexus Clinical Research	Chicago, IL
Richard M. Novak, MD	Regina Harden, Maria Schwarber, Michael Pacini, Rebeca Gansari, Margie Villarreal, Stephanie Martin, Michelle Lee, Richard Morrissy, , Taylor Ellis, Samuel Rene, Tara Cobbs, Claudia Preciado, Scott Borgetti, Maximo Brito, Olamide Jarrett, Mahesh Patel, Tracy Cable, Charity Ball, Maryann Holtcamp, Rodrigo Burgos, Sarah Michienzi, Emily Drwiega, Mikayla Johnson, Fischer Herald, Benjamin Ladner, Minseung Chu, Carolyn Dickens, Alfredo Mena Lora, Stockton Mayer, Andrea Wendrow, Habiba Sultana, Nanu Nunwar, David Chan, Marla Schwarber, Khandaker Anwar, Mahmood Ghassemi, Md Ruhul Amin, Doris Carroll, Rosa Valencia, Michelle Agnoli, Elena Llinas, Samuel Rene, Liam Morrissy, Adrian Raygoza, Addis Mekkonnen, Lisa Lindemann, Daniel Meslar, Karen Pacini, Corey Ringhisen, Amy Kennedy-Krage, Claudia Miller, Lorna Sanchez McCann, Gizelle Alvarez, Nia Moragne-Oneal, Nusirat Williams, Ian Feather, Nikki Griffith, Wardrick Nealon, Renyce Powell, Nila Safaeian, Monica Gingell, Diana Bahena, Gerald Beck, Brad Farrington, Rod Reyes, Monica Wilson, Juline Wondrasek, Kimberly Shapiro, Shannon Whitted, Victoria Roehl, Braulio Carrasco, Michael Chen, Olivia Murray, Yasiel Lacalle, Tessa Eckley, Anna Schluckebier, Kevin Cao, Elise DeBruyn	University of Illinois at Chicago - Project WISH	Chicago, IL
Paul Joseph Nugent, DO	Leonard Singer, Jennifer Jones, April Smith, Georgettea Geuss, Lana Ghomrawi, Christine Bennett, Norma Blevins, Linda Brotherton, Michele Byrd, Krista Doss, Victoria Holden, Christine Hull, Jean Montgomery, Nancy Cipollone, Savanah Torline, Brandon Brown, Meagan Thomas, Katie Ziska, Dana Sias, Hannah Wagner	Synexus Clinical Research	Cincinnati, OH
Jeffrey Scott Overcash, MD	Hanh Chu, Kia Lee, Karla Zepeda, John Rodriguez, Adam Prince, Yashveer Dubbula, Elizabeth Tomatsu Michael Voskanian, Crystle Rajania, Stephanie Ramirez, Claudia Camacho, Lauren Arnett, Kecia Darbeau, Ashley Smith, Kimberly Quillin, Cesar Ramirez, Daniel Robitaille, Erica Sanchez, Allie Davis, Michael Waters, Pat Kappen, Valerie Horne, Thao Vuong, Andrew Dennis, Nikki Abels, Dominique Panis, Richard McQuaid, Whitley Harbison, Erika Trujillo, Andrea Garcia, Jose Jacob Esparza, Carlos Vera, Raquel Taitingfong, Cathy Meza, He Pu, Jackielynn Smith, Shandel Odom, Zahira Nieves, Ashliegh Lindsay, Ariana Nasatka, Jose Cazarez, Nora Martinez, Angela Hunt, Antonio Delgado, Linda Vega, Angela Anorve, Erica Martinelli, Melania Riordan, Sylvia Lindholm, Gina Ciezkowski, Grecia Perez, Jacob Pineda,	Velocity Clinical Research, San Diego	La Mesa, CA

Principal Investigator	Study Team	Institution	Location
	Nathan Tyler, Ranya Salem, Amara Yilmaz, Jessica Gonzales, Zabrina Ruiz, Laura Castillo, Yajaira Contreras, Angelica Guzman, Makenna Orel, Jeffery Alvarez, Gordon Bovee, Roxana Ramirez, Joan Esquivel		
James Todd Peterson, MD	Christopher Mickelson, Madeline Maldonado, Alison Charlton, Ashley Bragg, Sean Hansen, Emily Wilcox, Colby Bostock, Megan Henry, Pam Iwasaki, Bradley Young, Katelyn Walker, Joy Nguyen, Lindsey Bevan, Megan Grimmett, Madeline Grote, Heather Littell, Natalie Bee, Alexander Clark, Shana Eborn, Susan Edwards, Dan Henry, Heather Jackson, Gerald Kelty, Issac Pena-Renteria, Jacqueline Rohrer, Jack Taylor, Brooke Barrick, Ty Henry, Anna Dansie, Kenadie Hamblin	J. Lewis Research	Salt Lake City, UT
Paul Pickrell, MD	Susan Bonner, Blaire Graham, Staci Taggart, Hussain Malbari, Tiffany Lemuz, Ethan Shotton, Andrew Bell, Megan Malek, David Pampe, Carol Ann Linebarger, Michelle Peterson, Brandi Chalman, John Luna, Elizabeth Santellanes, Christina Martinez, Lisa Johnson, Lisa Savage, Melissa Winn, Wendi McKenzie, Eileen Euperio, Stefanie Mott, Paul Menefee, Katie Caballero, Darrell O'Brien, Morgan Schulle, Kate Jurek, Olivia Hapanowicz	Tekton Research, Inc.	Austin, TX
Terry L. Poling, MD	Meenakshi (Kavya) Natesan, Patricia Contreras, Denise Hole, Avi Woods, Jill Hiebert, Melissa Burton, Olivia Eagleson, Laura Holz, Terri Ford, Cindy Thome, Terry D Klein, Gregory Greer, Diandra Henriques, Tracy R Klein, Thomas C Klein, Christa Shue, Gina Young, Brenna Sprout	Alliance for Multispecialty Research	Wichita, KS
Bruce G. Rankin, DO	Jennifer Dittman, Lora Parahovnik, Crystal Paccione, Melissa Hodges, Katina Marchione, Matt Maxwell, Any Dominy, Diana Toney, Andrea Marrafino, Laura Isbell, Leandro Fernandez, Claxton Copeland, Michelle Tutt, Adam VanDeusen, Kevin Feldman, Clark Mason, Tifany Huertas, Over Seijas, Jennifer Cline, Christian Beierschmitt, Ryan Hobbick, Jessica Gilliam, Jeanette de Leon, Iman Mencia, Daniel Layish, Vienna Bauer, Shatonia Fields, Albert Garcia, Carrie Rycort, Tasha Brocato, Marshall Nash, Samantha Watts, Amy Houck-Dominy, Angela Hammerle, Teresa Logsdon, Erika Wierzbicki, Taylor Martin, Ranie Hutchins, Fadhel Alyunis, Gail Lavine, Jeffery Hood, Robert Duran, Michelle Jones, Ginny McClanahan, Heather Jackson, Leandra Fernandez, Douglas Winter, Antonio Rivera, Amber Vasquez, Thais Truffa, Daniel Campbell, Grace Newcomb, Elizabeth Orlando, Steven Shinn, John Hill, Christina Isbell, Dhaneshwar Oomrow, Alicia Cevera	Accel Research Sites	DeLand, FL
Michele Diane Reynolds, MD	Jennifer Bashour, Robert Schmidt, Cynthia Mayeux, Uvoka Huffman, Lisa Nicholson, Jacklyn Newton, Lynn Yauch, Cathy Monroe, Kathleen Carty, Angelica Banks, Taylor Werner, Pamela Echols, Pauline Jackson, Chana Hines, Lorine Cook, Cristina Puig, Patrick Brooks, Jennifer Ruiz, Deanna Bowman, Ladina Garcia	Synexus Clinical Research	Dallas, TX
Rambod Rouhbakhsh, MD, MBA	John Johnston, Richard Calderone, Tasha Stevenson, Tameka Fortune, Brandi Pace, Adreanna Pou, Jerrica Sullivan, Yolanda Lewis, April Rouse, Tiffany Jefferson, Elizabeth Danford, Jeff Repper, Mason Boutwell, Alexycia Washington, Krista Hirth, Meagan Grabel	MediSync Clinical Research Hattiesburg Clinic	Petal, MS
Nadine Rouphael, MD	Renata Dennis, Tigisty Girmay, Michelle Wiles, Sharon Curate-Ingram, Lauren Hewitt, Alexis Ahonen, Mari Hart, Sarah Bechnak, Erin Carter, Lauren Nolan, Daniel Sans Graciaa, Geoffrey Kamau, Easton Beshears, Sy Tran, Mary Atha, Mary Bower, Ghina Alaaedine, Brandy Johnson, Jacob Usher, Eileen Osinski, Erin Scherer, C. Tae Stallworth, Stephanie Ramer, Rose Pope, Esther Park, Francine Dyer, Laura Clegg, Rebecca Gonzalez, Stacey Wheeler, Susan Rogers, Vy Ngo, Vanessa Soliman, Kristen Unterberger, Bernadine Panganiban, Christopher Huerta, Juton Winston, Ali Alvarez, Jianguo Xu, Colleen Kelley, Paulina Rebolledo, Nicholas Scanlon, Jessica Traenkner, Matthew Collins, Hollie Macenczak, Cassie Grimsely-Ackerley, Tiffany Lee, Amy Anderson, Michele Paine McCullough, Hannah Huston, Daniella Carter, Lisa Harewood, Srilatha Edupuganti, Varun Phadke, Mindee Adamson, Jeanne Allen, Debbie Bartenfeld, Lily Berz, Amy Cromwell, Sergio Cruz, Fred Ede, Monica Godfrey, Evan Gutter, Angelle Ijeoma, Sara Jo Johnson, Vinit Karmali, Dean Kleinhenz, Jennifer Kleinhenz, Alexandra Koumanelis, Maranda Leary, Tiraje Lester, Juliet Alise Morales, Shashi Nagar, Julia Paine, Dilshad Rafi Ahmed, Brittany Robinson, Amanda Rosner, Renee Silver, Trevor William Simon, Talib Sirajud-Deen, Damien Swearing, Maliya Tolbert, Pamela Turner, Chia Uzuegbunam, Claire Wan, Dongli Wang, Erika Wimberly, Jean Winter, Joy Winters, Yong Xu, Sha Yi	Emory University - Hope Clinic	Decatur, GA
Richard Rupp, MD	Amber Stanford, Megan Berman, Laura Porterfield, Gerianne Casey, Hala Ghoson, Doreen Jones, Michael Willig, Cori Burkett, Robert Cox, Amy McMahan, Diane Barrett, Kristin Pollock	University of Texas Medical Branch	Galveston, TX
Jamshid Saleh, MD	Matthew Miles, Rafael Lupercio, Vicky Martin, Marla Clark, Matthew Pohlmeyer, Ruba Zanaid, Veronica Blevins, Tara Ulberg, Carlyee Chambers, Marisol Corrales, Emily Crews, Mohamed Yassin, Sarah Sandberg, Frank Chen, Mandy Swanson	Paradigm Clinical Research Center	Redding, CA
John W. Sanders, MD, MPH	Stacy Harpe-Hall, Jesse Hopkins, Ann Schweppe, Jaymous Fayssoux, Kathryn Bender, James Peacock, Katharine Pearsall, Brandy Snyder, Deidre Knox, Megan Thorpe, Melissa Ellingson, Brittany Bundeff, Lisa Ashworth, Meredith Hiatt, Ritu Rathee, Stacy Woodliff, Brian Strittmatter, Amanda Wright, Daisy DeWeese-Gatt,	Wake Forest University Health Sciences	Winston Salem, NC

Principal Investigator	Study Team	Institution	Location
	Caryn Morse, John Williamson, Samantha Wheeler, Lori Whiteheart, Susan Donahue, James Lovette, Kaitlyn Van Leuvan, Kelly Ledbetter, Scott Chatterton, Julio Nasim, Amie Sidberry, Ashley Davis, Carter Noecker, Chie Hooker, Johanna Breenan, Sam Cable, Anna Bowman, Stephanie Boothe, Shea Overcash		
Howard I. Schwartz, MD	Carlos Valladares, Jocelyn Morrera, Yulexis Amestoy, Tori Wallenburg, Thelma Beltran, Terry Piedra, Monica Garces, Alexandra Galvis, Wanda Delgado, Catherine Casas, Lesly Miguel Sosa, Vivian Rosales, Jose Fernando Henriquez, Mikael Yaniz, Beatriz Rivera, Peter Ventre, Gabriella Huyke, Maria Companioni, Jessie De Vega, Brianna Gamez, Stephanie Diaz, James Jean-Mary, Americo Padilla, Nikita Notise, Yorlina Luquetta, Monifa Wilson-Morris, Kenia Gutierrez, Roilan Garcia, Karla Pentzke, Leyda Valentin, Lazara Novas, Marilein Camacho, Jazmin Henfield, Laymis Alvarez, Myriam Rosado, Maxine Bryant, Maria Pinero, Laura Raucci, Francisco Ramirez, Angelic Gamez, Mailin Perez, Yasmin Baddour, Hary Leon Joseph, Yaquelin De la Cruz, Dunia Torres, Rosaidaliz Carreira, Chanella Garcia, Surisaday Mederos, Jose Muniz, Karenda Plotka, Sara Gomez, Maria Soto, Cathy Cruz, Nelia Sanchez-Crespo, Jennifer Schwartz, Barbara Corral, Matthew Muniz, Dayana Deltejo, Ana Castro, Reem Hassan	Research Centers of America	Hollywood, FL
Nathan Segall, MD	Michelle Sowell, Nancy Levine, Erynn McKinley, Hannah Smith, Karen Hickson, Elizabet West, Patrizia Greene, Jon Finley, Mildred Stull, Susan Jones, Jennifer LeBrun, Pamela Talbott, Kwannda Whatley, Jeffrey Jones, Michelle Binns, Donna Toepfer, Cynthia Steele, Grace Newville, Gillian Waite, Cynthia Pinckney, Karen Yangapatty, Kiara Tyner, Kimberly Cobb, Kourtney Richardson	Clinical Research Atlanta	Stockbridge, GA
William Seger, MD	Kimberly Pullen, Jean Seignon, Anthony Kim, Mohammed Antwi, Allison Green, Lizzy Seger, Elizabeth Boydston, Abdur Rafay Qadri, Deborah Devlin, Tasha Todd, Oluwatosin Akingbala, Alma Guel, Tisha Davis, Melody Dufrene, Samantha Loudermilk, Virginia Loudermilk, Crystal Starr, John Villegas, Ben Seger, Katherine Hollie	Benchmark Research	Fort Worth, TX
Neil Parmanand Sheth, MD	Kenneth Stell, David Beckett, Enitt Gonzalez, Donna McGunigal, Amanda Burns, Nancy Wood, Shelley Miceli, Christina Avila, Rebecca Baker, Laura Vigliotti, Sarah Kading, Samer Salama	Synexus Clinical Research	Glendale, AZ
William B. Smith, MD	Richard L Gibson, Jennifer Winbigler, Elizabeth Parker, Madison Watts, Suzann Cloninger, Talya Thomas	Alliance for Multispecialty Research	Knoxville,TN
Joel Solis, MD	Martha Carmen Medina, Xavier Morales, Hank Heller, Blake Torrence, Joanna Gurrola-Mahoney, Cynthia Bueno, Heather Holloway, Irving Salinas, Joel Perez, Paola Garcia, Erica Canales, Blanca Urbina, Brancisilio Gutierrez, Carolina Cantu, Chelsea Vargas, Cindy Vasquez, Cody McIntire, Gabriela Gutierrez, Hugo Sosa, Irvin Munoz, Jessica Estrada, Jonna Lopez, Kaegan Knox, Mirella Melendez, Natalia Valle, Natalie Echavarria, Nicole Litton, Amber Victor, Nancy Torrence, Madhu Shreya, Mathew Maran, Asfak Alam, Westly Keating, Tara Green, Devora Torrence, Gerardo Sedas, Shruti Konda, Prem Jangam, Mario Echavarria, Alejandro Silva, Anne McNulty, Daniel Contreras, Daniel Gomez, Edgar Garcia, Elizabeth Weber, Luis Lopez, Samuel Ramirez, Kayla Lopez, Pedro Penalo, Angel Salinas, Jaime Solis, Shannon Moyer, Aryana Ibarra, Guadalupe Gurrola, Jenna Anastasiades, Uchechi Ehiemua, Sara Solorzano	Centex Studies, Inc.	McAllen, TX
Stephen A. Spector, MD	Amaran Moodley, Jill Blumenthal, Baharin Abdullah, Christina Addington, Juan Carlos Alcantar, Deyna Arellano, Bernadette Cale, Brendan Costello, Tammelita Cotlon-Pineda, Fanny Delebecque, Karen Deutsch, Aram Dimayuga, Son Do, Yasmeen Esshaki, Aileen Everhart, Cindy Ewing, Veronica Figueroa, Medardo Gaytan, Crystal Groom, Carolyn Hernandez, Heather Huitema, Benjamin Hull, Sylvia Isaac, Jaclyn Jaskowiak, Cindy Knott, Leander Lazaro, Thuan Le, Megan Loughran, Michelle Madey, Rosalva Martha-Patten, Colleen McLellan, Jeff Ledford-Mills, Asami Mimura, Patty Moraes, Jennifer Morales, Jessica Nasca, Phirum Nguyen, Marielys Padilla-Martinez, Dennis Perpetua, Mike Pizza, Shannon Ransom, Emily Rizo, Carlos Rojas, Thaine Ross, Marie Sagrado, Eugene Sato, Lisa Stangl, Ji Sun, Nancy Tang, Mina Trivedi, Rodney Trout, Donna Voss, Lindsey Woronicz	University of California, San Diego	La Jolla, CA
Cynthia Becher Strout, MD	Rica Santiago, Yvonne Davis, Patty Howenstine, Alison Bondell, Jaime Robertson, Anissa Moussa, Geronimo Feria Garzon, Sierra Bennett, Marlena Petrie	Coastal Carolina Research Center	Mount Pleasant, SC
Shobha Swaminathan, MD	Amesika Nyaku, Tilly Varughese, Rondalya Deshields, Michelle L DallaPiazza, Elise Lewis, Jennifer Punsal, Mario Portilla, Malithi Desilva, Christina Daliani, Susana Rivera, Aidan Ziobro, Andressa Rebellatto, Brian Murloy, Christina Ninan, Ernest Pianim, Eunice Wang, Merit Henen, Muhammad Usman, Rebecca Kim, Shiao Wang, Gener Eric Cruz, Bethany Birago, Joyell Arscott, Dina Meawad, Christie Lyn Costanza, Francesca Escaleira, Zoraida Cruz-Barahona, Jared Khan, Valeria Cadorett, Jamir Tuten, Travis Love, Eric Asencio, Sukhwinder Singh	Rutgers New Jersey Medical School	Newark, NJ

Principal Investigator	Study Team	Institution	Location
Ramy Joseph Toma, MD	Olivia Graves, Josiah Robinson, Patricia Hammonds, Lana Ghomrawi, Kara Quinnelly, Shaun O'Conor, Michael Lambe, Rachell Stewart, William Kirby, Pink Folmar, Rachel Culbreth, Heidi Leblanc, Julie McDaniel, Rian Montgomery, Andrea Woodle, Samantha Williams, Hunter Russell, Shereen Lowe, Maureen Mayer, Hollis Ryan, Elaine Reese	Synexus Clinical Research	Birmingham, AL
Timothy P. Vachris, MD	Mark Hutchens, Stephen Daniels, Margaret Wells, Sandra Clancy, Rebecca Martinez, Jessica Buot, Merissa Daugherty, Julie Hamilton, Kimberly Hernandez, Ashli Alejandro, Amy Collins, Monique Gawlik, Patricia Johnson, Maria Moreno, Ashley Washington, Tina Rountree, Daniel Dore, Ravi Davuluri, Ashlee Brunaugh, Jorge Martinez, James Hermon, Vianai Carreno, Mia Rountree, Colleen Coelho	Optimal Research	Austin, TX
Keith William Vrbicky, MD	Charles Harper, Chelsie Nutsch, Wendell Lewis III, Cathy Laflan, Linden DeBoer, Kayla Andal, Misty Appeldorn, Jenniger Grebe, Russell Herstein, Catherine King, Samantha Wieseler, Alisha Kiepke, Christy Lee, Kelsey Kelley, Kelli James, Ashley Frisch, Courney Green, Taysha Hingst, Jeni Hoppe, Kimber Breeden, Debra Gabrielson, Ginny McNew	Meridian Clinical Research	Norfolk, NE
Larkin T. Wadsworth III, MD	Ashley Dale, Christy Schultz, Rebecca Munsch, Anya Penly, Liz Garner, Stephanie Tesson, George Cherniawski, Angie Kean, Dan Reed, Courtney Kubiak, Maureen Dempsey, Heather Cherniawski, Breanna Galibert, Kristin Branson, Laura Hartupee, Karen Knapp, Horacio Marafioti, Lyly Dang, Jennifer Berry, Lauren Clement, Megan Dandurand	Sundance Clinical Research	St. Louis, MO
Jordan L. Whatley, MD	Patricia Whatley, Christopher Dedon, Anika Payne, Amie Shannon, Kristen Losavio, Nicole Harrell, Mary Margaret Dobson, Lindsey Hall, Chaney Bennett, Crystal Rowell, Mimi Dimmick, Amy Thomassie, Kimber Breeden, Cody LaFleur, Makaylea Truitt, Taryn Collett, Emily Best, Alexandra Caillouet,	Meridian Clinical Research	Baton Rouge, LA
Judith White, MD	Amy Edridge, Chelsea Montalvo, Eugenia Clark, Lisa Russell, Zahra Somji, Lesli Leimer, Robert Meyer, Christine Murphy, Prity Patel, Sejal Patel, Ruben Moliere, Samantha Merveillard, Yarnick Mirjah, Bryn Walls, Joey Cruz, Aaron Cooper, Jessica Bienaime, Ashley Gilcrist, Alisa Petit, Tyler Knightly, Kimberly Stokes, Christina Rosario, Talhia Matos, Ilona Boggs, Nicholas Weber, Felix Busot, Linda Colon, Heather Gillenwater, Cristina Kaplun, Melissa Caputi, Shayna Siplin, Daminee Shah, Samuel Martin, Alexis Waldorf, Vihar Upadhyay, Adolfo Henriquez, Saskia Singh, Maria Roberts, John Caporelli, Shirley Salvador, Quevina Scarver, Vanessa Garcia, Taylor Moore, Jayasen Singh, Curshinda Galvin-Burch, Mary Kesner, Jasmin Gil, Shay Gray, Steven Monsegur, Michele Steinmetz, Michael Lambe, Heather Powell, Sandra Torres, Shaban Katbeh, Taylor Wilson	Synexus Clinical Research	Orlando, FL
Priyantha N. Wijewardane, MD	Natalie Johnson, Martha Evans, Sondra Wright, Richard Pellegrino, Lastida Burns, Natasha Williams, Haylee Rowe, Kayla Graham, Amanda Horn, Eric Bravo, Jeffrey Thessing, A. Michele Maxwell, Amy Cooper, Lauren Evans, Tonya Cato, Haylee Tucker, Lesa Gann, Hannah Jones, Amanda May, Tiffany Walker, A. LeiAn Diaz, Laura Khalil, Lydia Purcell, Timothy Campbell, Charlotte Garcia-Velez, Andrea Scarborough, Beatrice A. Miller, Keith Bracy, Aujania Thompson, Cassandra Johnson, Krishana Day, Freddie Hicks, Jamie Pettus	Baptist Health Center for Clinical Research	Little Rock, AR
Barton G. Williams, MD	Flo Abbott, Nicole Burton, Alice Cipollini, Madison Croucher, Philip Dattilo, Erin Harrelson, Kelsey Heston, James Ingram, William H Jones, Karla Lane, Brandy Lowman, Evan Lucas, Megan Marles, Morgan Mathis, Angie Northcott, Clyda Pasquantonio, Alyssa Valente, Ciara Winders, Stephanie Graham	Trial Management Associates	Wilmington, NC
Marcus J. Zervos, MD	Paul Kilgore, Mayur Ramesh, Jelena Verkler, Pardeep Pabla, Andrew Clark, Katrina Williams, Dee Dee Wang, Beverley Duthie, Samia Arshad, Alandra White, Anna Kern, Ashley Mattern, Bilqis Mosed, Dana Parke, Doreen Dankerlui, Dragana Spasevska, Hanah Woods, Helina Misikir, Howard Klausner, Janay Scott, Jessica Heinonen, John Zervos, Joseph Miller, Kate Zenlea, Kristin Eis, Marissa Vasquez, Maurice Slaughter, Meaghan Flynn, Michael Garcia, Michelle Sankah, Nina Paeilli, Philip Benson, Robert Devore, Stevanya Baho, Tony Eljallad, Tyler Prentiss, Yaman Ahmed, Sharon Mathys, Linda Kaljee, Jeffrey Van Laere, Claudia Hanni, Hassan Zafar, Mona Desai, Gina Maki, Mary Perri, Dora Vager, Shannon Thomas, Autumn Robinson, Isis Hamilton, Sonia Eliya, Jehan Jazrawi, Biljana Popovic, Sharon Zahul, Joshua Ruzzin, John Laguio, Ali Mathena, Bobby Cook Jr., Marlene Hesler, Rochelle Fleming, Terria Minniefield, John Simons, Sherese Henderson, Ashley Hopkins, Rebecca McFarlane, Raeshell Carson, Jonathan Williams, Katherine Reyes, Erica Herc, Indira Brar, Mayur Ramesh, John McKinnon, Lacquis Duncan, Tim Asmar, Margaret Beyer, Kaleem Chaudhry, Madison Lee, Jo-Ann Rammal, Karthik Sridasyam, Siddesh Veer, Angelique Buluran, Kimberlyn Lott, Jeremiah Rooker, Alayna Wilder, Kathleen Wilson, Allison Weinmann, Hassan Mourtada	Henry Ford Health System	Detroit, MI

United States Government (USG)/Coronavirus Prevention Network (CoVPN) Biostatistics Team
(PubMed listed, and ordered alphabetically by institution affiliation)

Affiliation	Team Members
Biomedical Advanced Research and Development Authority (BARDA), Washington, DC	Di Lu, James Zhou
Department of Biostatistics and Bioinformatics, Rollins School of Public Health, Emory University	David Benkeser
Vaccine and Infectious Disease Division, Fred Hutchinson Cancer Research Center, Seattle, WA	Jessica Andriesen, Bhavesh Borate, Lindsay N. Carpp, Andrew Fiore-Gartland, Youyi Fong*, Peter B. Gilbert*, Ying Huang*, Yunda Huang, Ellis Hughes, Ollivier Hyrien, Holly E. Janes*, Michal Juraska, Yiwen Lu, April K. Randhawa, Brian Simpkins, Brian D. Williamson, Lars W.P. van der Laan, Chenchen Yu
Biostatistics Research Branch, NIAID, NIH, Bethesda, MD	Michael P. Fay, Dean Follmann, Martha Nason
Division of Biostatistics, School of Public Health, University of California, Berkeley, CA	Nima S. Hejazi
Department of Biostatistics, University of Washington, Seattle, WA	Marco Carone, Kendrick Li, Wenbo Zhang
Department of Statistics, University of Washington, Seattle, WA	Alex Luedtke
Department of Population Health Sciences, Weill Cornell Medical College, New York, New York	Iván Díaz

*YF, PBG, YH, and HEJ are also affiliated with the Department of Biostatistics, University of Washington, Seattle, WA. PBG is also affiliated with the Public Health Sciences Division, Fred Hutchinson Cancer Research Center, Seattle, WA.

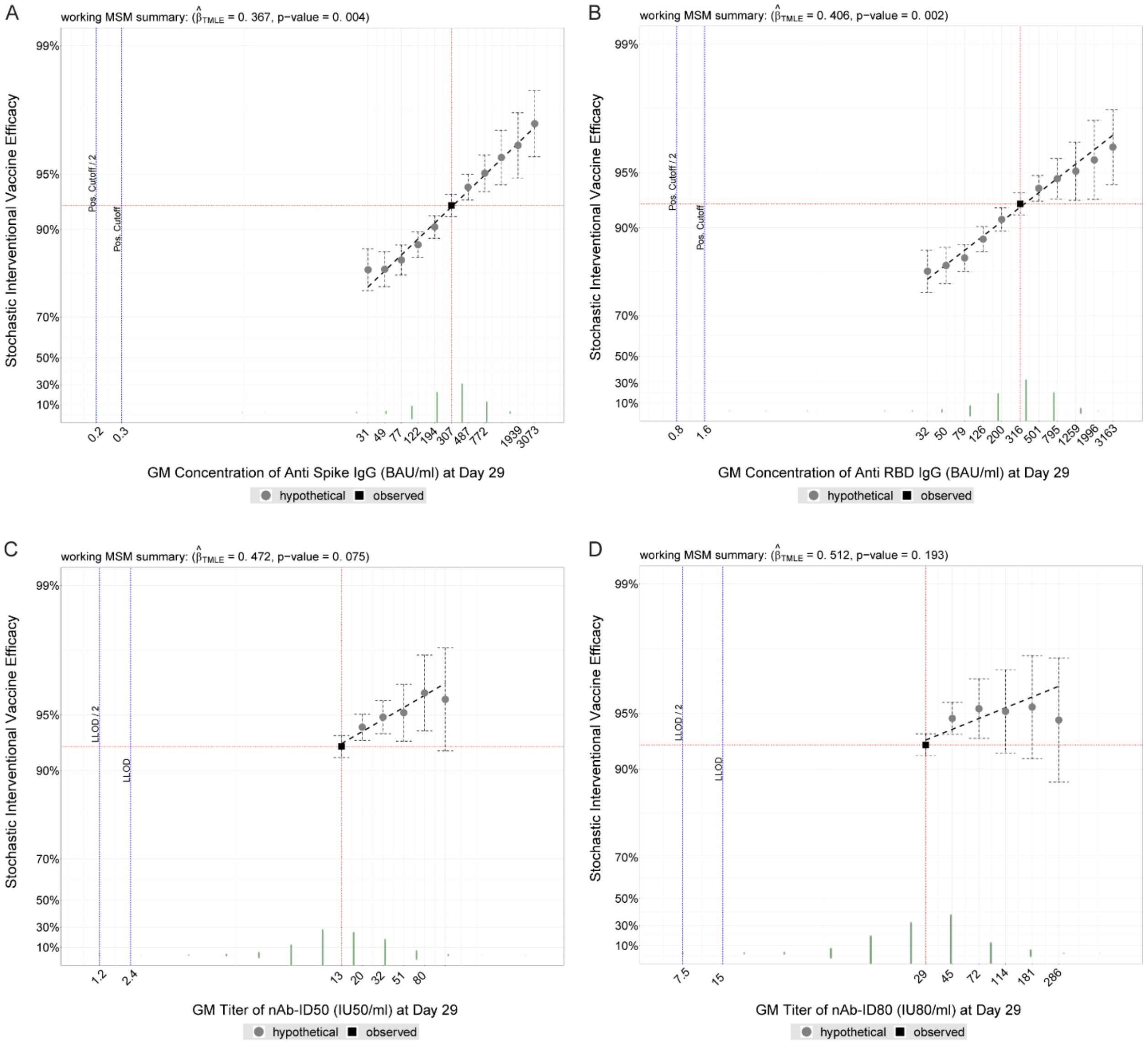

Figure S1. Stochastic interventional vaccine efficacy (SVE) estimates against COVID-19 with hypothetical shifts in geometric mean D29 antibody marker level. SVE, with 95% confidence intervals, for D29 (A) spike IgG, (B) RBD IgG, (C) nAb-ID50, or (D) nAb-ID80, estimated using the method in Hejazi et al.[1] The y-axis plots the estimated vaccine efficacy (VE) for a vaccine that elicits hypothetical D29 geometric mean value indicated on the x-axis. BAU, binding antibody units; ID50, 50% inhibitory dilution; ID80, 80% inhibitory dilution; IU, international units; LLOD, lower limit of detection; nAb, neutralizing antibody. The vertical red line corresponds to the geometric mean concentration or titer in the COVE study population (baseline negative per-protocol vaccine recipients in the immunogenicity subcohort) and the horizontal red line corresponds to the estimated VE in COVE (follow-up time period from 7 to 126 days post D29) at a shift of 0, i.e., the observed marker level.

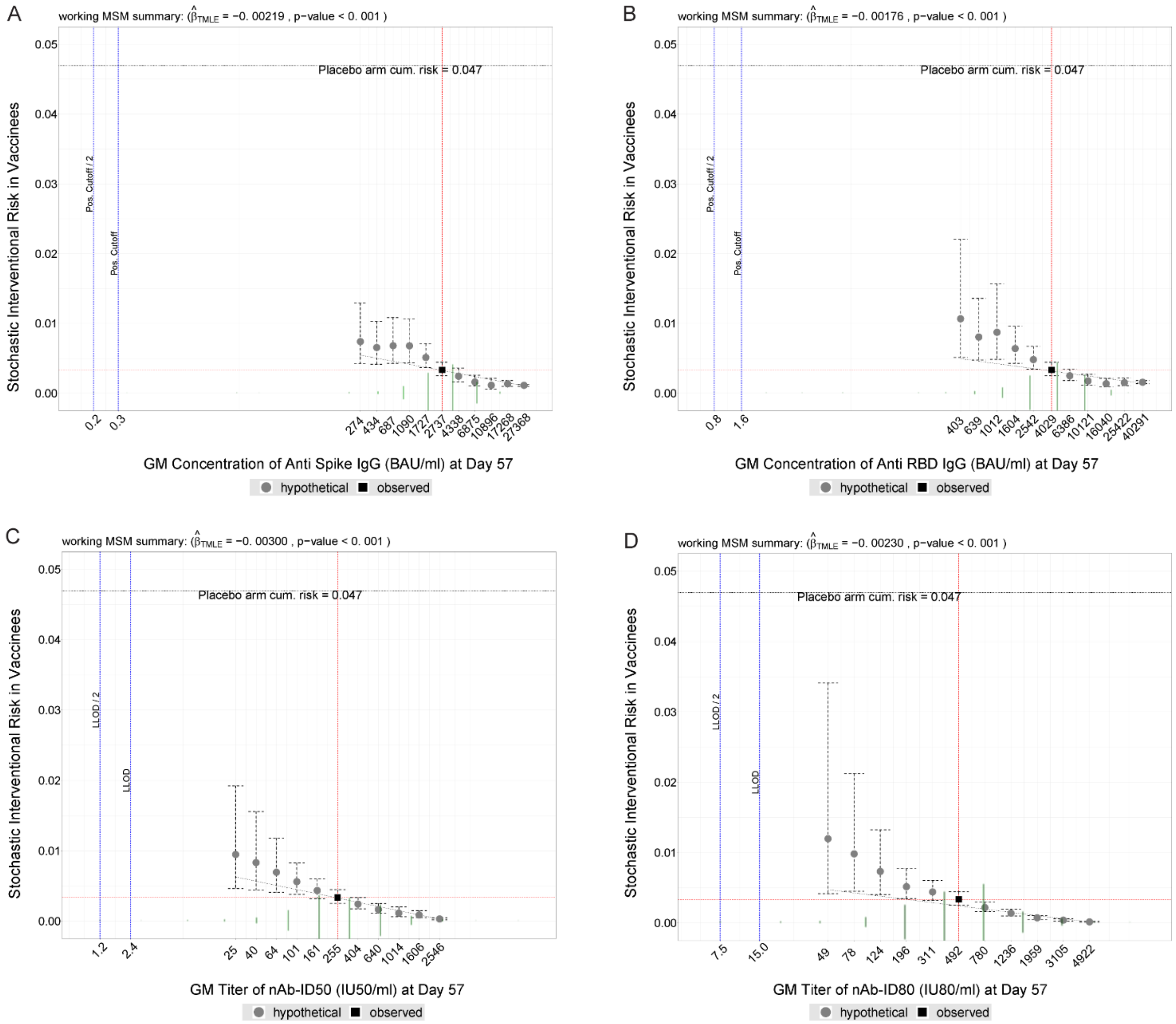

Figure S2. Estimates of absolute COVID-19 risk of baseline negative per-protocol vaccine recipients under different hypothetical shifts in geometric mean D57 antibody marker level: (A) spike IgG, (B) RBD IgG, (C) nAb-ID50, or (D) nAb-ID80. The follow-up time period was 7 to 100 days post D57. Note that the absolute risk estimates shown are simply the numerators of the stochastic interventional vaccine efficacy estimates shown in Figure 1. BAU, binding antibody units; ID50, 50% inhibitory dilution; ID80, 80% inhibitory dilution; IU, international units; LLOD, lower limit of detection; nAb, neutralizing antibody. The vertical red line corresponds to the geometric mean concentration or titer in the COVE study population (baseline negative per-protocol vaccine recipients in the immunogenicity subcohort) and the horizontal black dotted line corresponds to the estimated absolute COVID-19 risk in baseline negative per-protocol placebo recipients (follow-up time period from 7 to 100 days post D57).

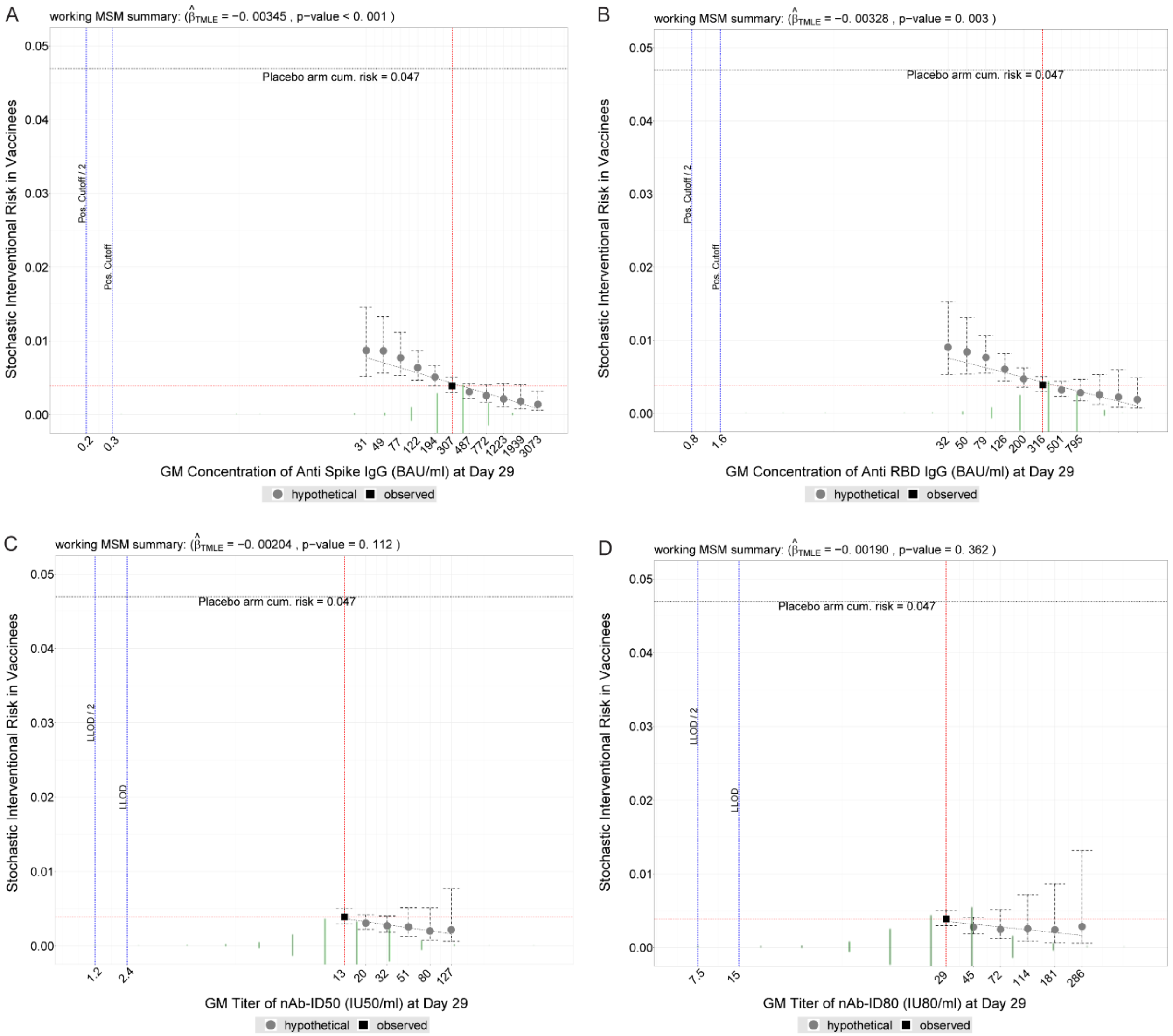

Figure S3. Estimates of absolute COVID-19 risk of baseline negative per-protocol vaccine recipients under different hypothetical shifts in geometric mean D29 antibody marker level: (A) spike IgG, (B) RBD IgG, (C) nAb-ID50, or (D) nAb-ID80. The follow-up time period was 7 to 126 days post D29. Note that the absolute risk estimates shown are simply the numerators of the stochastic interventional vaccine efficacy estimates shown in Figure S1. BAU, binding antibody units; ID50, 50% inhibitory dilution; ID80, 80% inhibitory dilution; IU, international units; LLOD, lower limit of detection; nAb, neutralizing antibody. The vertical red line corresponds to the geometric mean concentration or titer in the COVE study population (baseline negative per-protocol vaccine recipients in the immunogenicity subcohort) and the horizontal black dotted line corresponds to the estimated absolute COVID-19 risk in baseline negative per-protocol placebo recipients (follow-up time period from 7 to 126 days post D29).

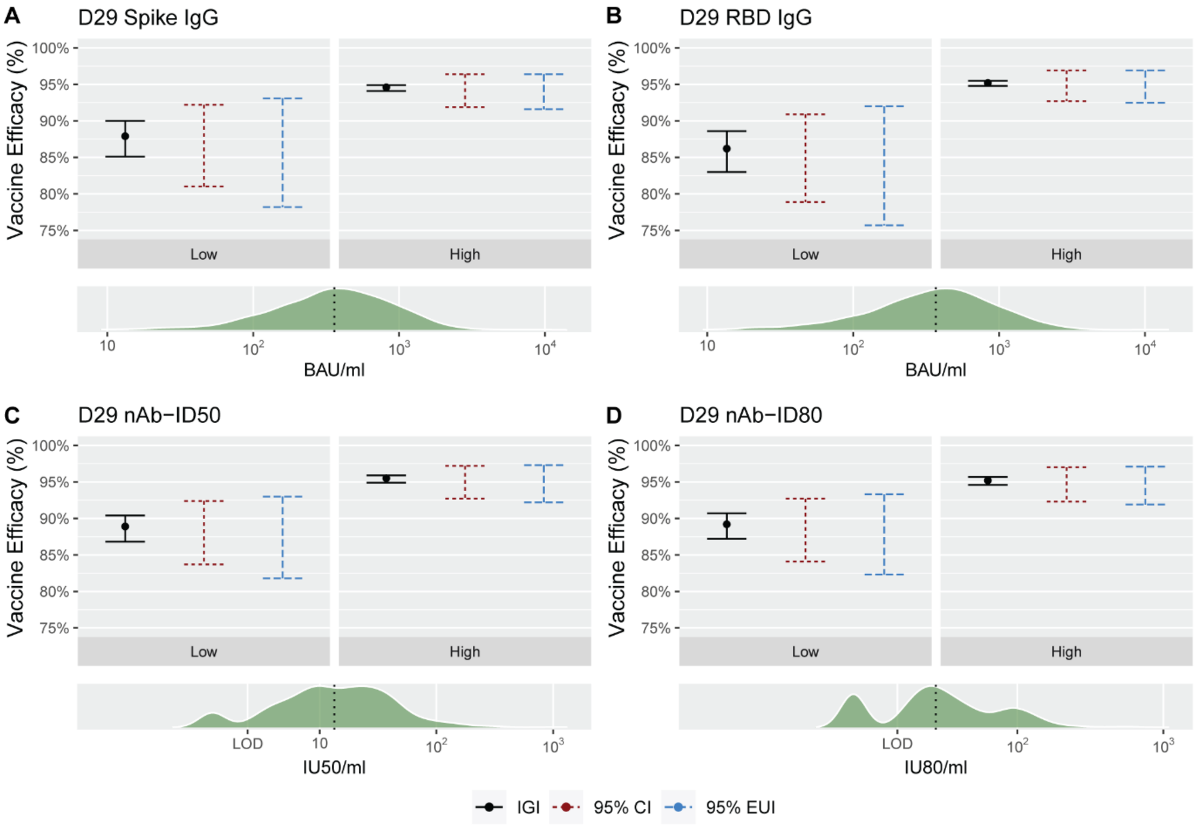

E

		Cut Point	For RR Ratio = [1 − VE(Low)]/[1 − VE(High)]<br>Point Estimate	IGI	95% CI	95% EUI
A)	D29 Spike IgG	204 BAU/ml	2.24	1.71, 2.94	1.24, 4.05	1.04, 4.84
B)	D29 RBD IgG	204 BAU/ml	2.90	2.21, 3.81	1.61, 5.22	1.34, 6.24
C)	D29 nAb-ID50	10 IU50/ml	2.45	1.87, 3.22	1.34, 4.49	1.12, 5.36
D)	D29 nAb-ID80	24 IU80/ml	2.24	1.71, 2.95	1.23, 4.08	1.03, 4.87

Figure S4. Binary principal surrogate vaccine efficacy (VE) against COVID-19 by D29 antibody marker greater than vs. less than or equal to the designated cut-point (median value). The black dot in each panel corresponds to the VE estimate for the relevant D29 antibody marker subgroup (Low or High) for (A) Spike IgG, (B) RBD IgG, (C) nAb-ID50, or (D) nAb-ID80 when **β** sensitivity parameters are set to zero. The vertical black line denotes the ignorance interval (IGI) when **β** sensitivity parameters range from log(0.75) to –log(0.75), the vertical red dashed line denotes the 95% confidence interval (CI) when **β** sensitivity parameters are set to zero, and the vertical blue dashed line denotes the 95% estimated uncertainty interval (EUI) when **β** sensitivity parameters range from log(0.75) to –log(0.75). The green histogram on each lower panel denotes the distribution of the D29 antibody marker, with the vertical black dashed line placed at the cut-point separating a Low D29 antibody marker response from a High D29 antibody marker response. This cut-point was the median marker value in baseline negative per-protocol vaccine recipients in the immunogenicity subcohort. (E) For each antibody marker, cut-point, relative risk (RR) ratio point estimate, IGI, 95% CI, and 95% EUI. RR ratio = (1-VE(0))/(1-VE(1)). BAU, binding antibody units; ID50, 50% inhibitory dilution; ID80, 80% inhibitory dilution; IU, international units; nAb, neutralizing antibody.

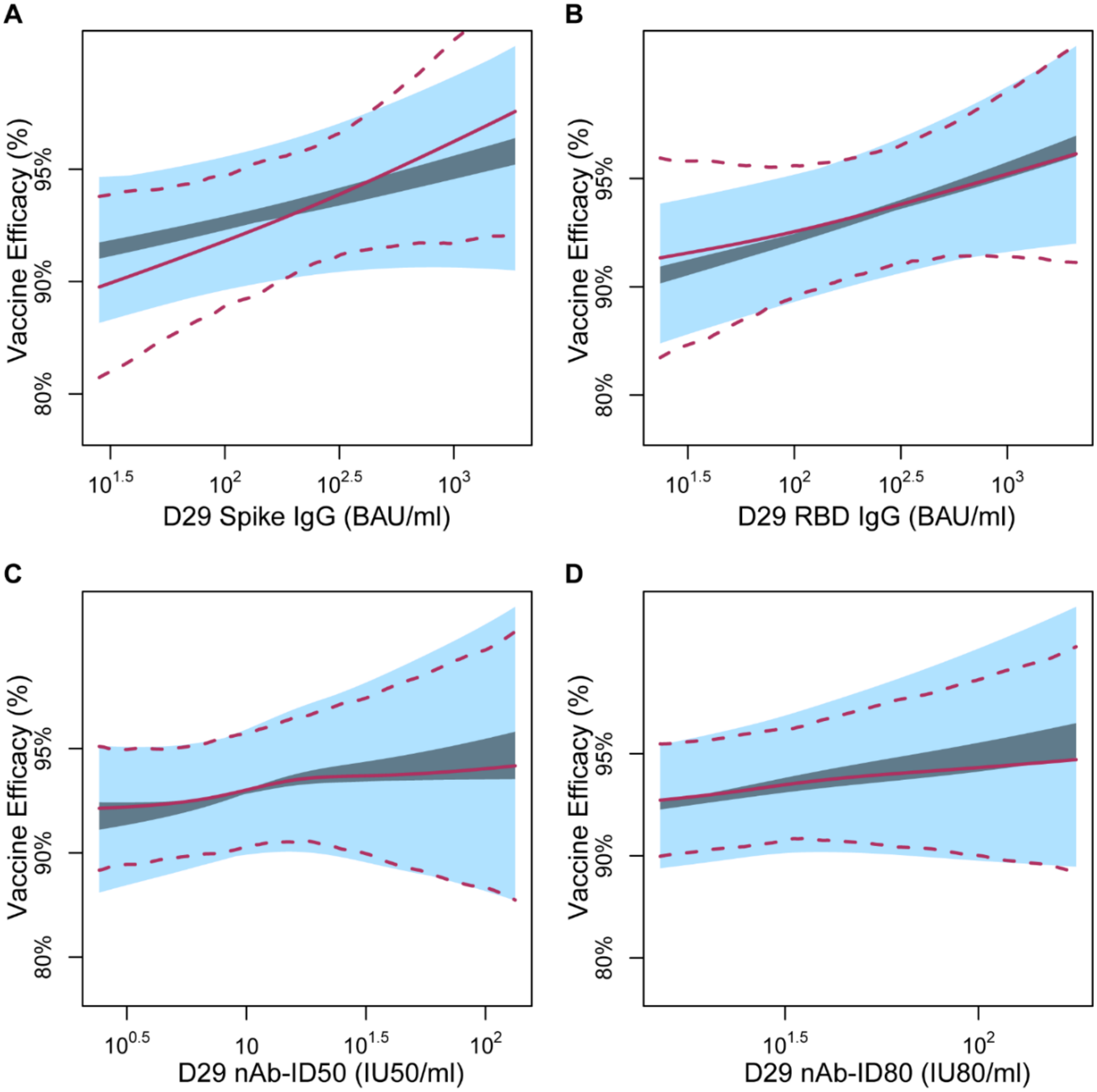

Figure S5. Continuous principal surrogate vaccine efficacy against COVID-19 by D29 marker level, with ignorance intervals (dark blue) and 95% estimated uncertainty intervals (light blue) under the No Early Harm (NEH) shown for the sensitivity parameter **β** assumed to fall in the range [–log(4), 0]. Results are shown for (A) Spike IgG, (B) RBD IgG, (C) nAb-ID50, or (D) nAb-ID80. In each panel, the solid and dashed lines are the estimated VE curve and 95% perturbation confidence intervals with the Equal Early Clinical Risk (EECR) assumption. The curves are plotted over the marker range of the 2.5th to 97.5th percentile (Spike IgG: 28.3 to 1852.8 BAU/ml, RBD IgG: 23.4 to 2103.4 BAU/ml, nAb-ID50: 2.4 to 133 IU50/ml, nAb-ID80: 15 to 178.7 IU80/ml). BAU, binding antibody units; ID50, 50% inhibitory dilution; ID80, 80% inhibitory dilution; IU, international units; nAb, neutralizing antibody.

Table S1. Principal surrogate correlates of vaccine efficacy (VE) results by Gilbert et al. method[2] for High (above median) vs. Low (below median) Day 29 antibody marker vaccinated subgroups under the No Early Harm (NEH) assumption with sensitivity analysis scenarios*

		**VE(0)**		**VE(1)**		**RR Ratio = (1-VE(0)) / {1-VE(1)}**	
		**Low Marker Vaccine Subgroup**		**High Marker Vaccine Subgroup**		**Relative Risk Ratio**	
Marker	Sens	**Ignorance Interval**	**95% Estimated Uncertainty Interval**	**Ignorance Interval**	**95% Estimated Uncertainty Interval**	**Ignorance Interval**	**95% Estimated Uncertainty Interval**
D29 Spike	None	(0.88, 0.88)	(0.81, 0.92)	(0.95, 0.95)	(0.92, 0.96)	(2.24, 2.24)	(1.24, 4.05)
D29 Spike	Med	(0.85, 0.90)	(0.78, 0.93)	(0.94, 0.95)	(0.92, 0.96)	(1.71, 2.94)	(1.04, 4.84)
D29 Spike	High	(0.80, 0.92)	(0.70, 0.95)	(0.93, 0.95)	(0.91, 0.97)	(1.16, 4.34)	(0.67, 7.15)
D29 RBD	None	(0.86, 0.86)	(0.79, 0.91)	(0.95, 0.95)	(0.93, 0.97)	(2.90, 2.90)	(1.61, 5.22)
D29 RBD	Med	(0.83, 0.89)	(0.76, 0.92)	(0.95, 0.96)	(0.92, 0.97)	(2.21, 3.81)	(1.34, 6.24)
D29 RBD	High	(0.77, 0.91)	(0.67, 0.94)	(0.94, 0.96)	(0.91, 0.97)	(1.50, 5.61)	(0.87, 9.22)
D29 nAb-ID50	None	(0.89, 0.89)	(0.84, 0.92)	(0.95, 0.95)	(0.93, 0.97)	(2.45, 2.45)	(1.34, 4.49)
D29 nAb-ID50	Med	(0.87, 0.90)	(0.82, 0.93)	(0.95, 0.96)	(0.92, 0.97)	(1.87, 3.22)	(1.12, 5.36)
D29 nAb-ID50	High	(0.83, 0.92)	(0.76, 0.95)	(0.94, 0.96)	(0.91, 0.98)	(1.26, 4.74)	(0.71, 7.88)
D29 nAb-ID80	None	(0.89, 0.89)	(0.84, 0.93)	(0.95, 0.95)	(0.92, 0.97)	(2.24, 2.24)	(1.23, 4.08)
D29 nAb-ID80	Med	(0.87, 0.91)	(0.82, 0.93)	(0.95, 0.96)	(0.92, 0.97)	(1.71, 2.95)	(1.03, 4.87)
D29 nAb-ID80	High	(0.83, 0.92)	(0.77, 0.95)	(0.93, 0.96)	(0.90, 0.97)	(1.15, 4.34)	(0.65, 7.18)

None: β sensitivity parameters β_2, β_3, β_4 set to zero
Med: β sensitivity parameters β_2, β_3, β_4 ranging from log(0.75) to –log(0.75)
High: β sensitivity parameters β_2, β_3, β_4 ranging from log(0.5) to –log(0.5)

Table S2. Principal surrogate correlates of vaccine efficacy results by Huang, Zhuang, and Gilbert method[3] for D29 antibody marker at various levels under the No Early Harm (NEH) or Equal Early Clinical Risk (EECR) assumption.

			Vaccine Efficacy (S_alpha)						
Marker	Assumption		alpha=0.025	0.05	0.1	0.5	0.9	0.95	0.975
D29 IgG Spike		Concentration (BAU/ml)	28.3	53.2	91.5	358.3	1112.8	1386.6	1852.8
	EECR	Estimate (%)	89.7	91	92	94.4	95.9	96.2	96.5
		CI (%)	(81.9, 94.1)	(85.6, 94.4)	(87.9, 94.8)	(91.7, 96.2)	(92.2, 97.9)	(92.4, 98.1)	(92.6, 98.4)
	NEH	IGI (%)	[91.3, 92.1]	[92, 92.7]	[92.6, 93.2]	[93.9, 94.5]	[94.8, 95.5]	[94.9, 95.7]	[95.1, 95.9]
		EUI (%)	(87.1, 94.8)	(88.4, 95)	(89.3, 95.3)	(90.7, 96.3)	(90.8, 97.3)	(90.8, 97.4)	(90.7, 97.7)
D29 IgG RBD		Concentration (BAU/ml)	23.4	47.6	84.7	365.1	1185.9	1501.4	2103.4
	EECR	Estimate (%)	91.7	92.3	92.8	94.2	95.3	95.5	95.7
		CI (%)	(84.3, 95.6)	(86.9, 95.5)	(88.9, 95.3)	(91.5, 96.1)	(91.7, 97.3)	(91.6, 97.5)	(91.4, 97.9)
	NEH	IGI (%)	[90.2, 91.2]	[91.4, 92.1]	[92.2, 92.7]	[94.1, 94.4]	[95.2, 95.7]	[95.4, 95.9]	[95.7, 96.2]
		EUI (%)	(85.6, 94.1)	(87.5, 94.6)	(88.7, 95)	(91, 96.3)	(92.1, 97.4)	(92.2, 97.6)	(92.4, 97.9)
D29 nAb-ID50		Titer (IU50/ml)	2.4	2.4	3.1	13.4	48.7	79.1	133
	EECR	Estimate (%)	92.6	92.6	92.6	93.7	94.1	94.2	94.4
		CI (%)	(88.8, 95.1)	(88.8, 95.1)	(89.2, 95)	(90.7, 95.7)	(88.9, 96.8)	(87.8, 97.3)	(86.3, 97.7)
	NEH	IGI (%)	[91.4, 92.8]	[91.4, 92.8]	[91.7, 92.8]	[93.5, 93.9]	[93.8, 94.8]	[93.9, 95.1]	[93.9, 95.5]
		EUI (%)	(87, 95.1)	(87, 95.1)	(87.6, 95.1)	(90.1, 96)	(88.6, 97.2)	(87.7, 97.6)	(86.3, 98)
D29 nAb-ID80		Titer (IU80/ml)	15	15	15	27.6	107.3	133.8	178.7
	EECR	Estimate (%)	93.1	93.1	93.1	93.7	94.5	94.6	94.8

	CI (%)	(90,95.3)	(90,95.3)	(90,95.3)	(90.8,95.7)	(89.9,97.1)	(89.5,97.3)	(88.8,97.6)
NEH	IGI (%)	[92.7,93.1]	[92.7,93.1]	[92.7,93.1]	[93.4,93.9]	[94.4,95.4]	[94.6,95.6]	[94.9,95.9]
	EUI (%)	(89.1,95.3)	(89.1,95.3)	(89.1,95.3)	(90,96)	(89.6,97.6)	(89.4,97.9)	(89.2,98.2)

CI, 95% confidence interval; EUI, 95% estimated uncertainty interval; IGI, ignorance interval. alpha = percentile of marker in vaccine recipients.

Table S3. Numbers of per-protocol baseline negative vaccine recipients with antibody markers measured and included in each of the Day 29 and Day 57 correlates analyses, for each of the four immunoassays. Adapted from Benkeser et al.[4]

	**Day 29 Correlates Analyses**		**Day 57 Correlates Analyses**	
**Antibody Marker**	Cases	Non-Cases	Cases	Non-Cases
Binding antibody to Spike (Spike IgG)	46	1005	36	1005
Binding antibody to RBD (RBD IgG)	46	1005	36	1005
Neutralizing antibody ID50 titer (nAb-ID50)	46	1005	36	1005
Neutralizing antibody ID80 titer (nAb-ID80)	46	1005	36	1005

Cases for Day 29 marker correlates analyses (Intercurrent cases + Post Day 57 cases) are baseline SARS-CoV-2 negative per-protocol vaccine recipients with the symptomatic infection COVID-19 primary endpoint diagnosed starting 7 days after Day 29 through the end of the blinded phase. Cases for Day 57 marker correlates analyses (Post Day 57 cases) are baseline SARS-CoV-2 negative per-protocol vaccine recipients with the symptomatic infection COVID-19 primary endpoint diagnosed starting 7 days after Day 57 through the end of the blinded phase. The last COVID-19 endpoint within the blinded phase occurred 100 days after Day 57.

For a comprehensive visualization of flow of participants from enrollment through to inclusion in this table, please see Figure S2 in Gilbert et al.[5]

As in Baden et al.[6] and El Sahly et al.,[7] the symptomatic infection COVID-19 primary endpoint was defined by at least two systemic symptoms (temperature ≥38°C, chills, myalgia, headache, sore throat, or new olfactory or taste disorders), or at least one respiratory sign or symptom (cough, shortness of breath, or clinical or radiologic evidence of pneumonia), and were confirmed by positive SARS-CoV-2 reverse-transcriptase–polymerase-chain-reaction (RT-PCR) assay of nasopharyngeal swab, nasal, or saliva samples.

Table S4. Calibration factors for expressing pseudovirus neutralizing antibody assay readouts in International Units.

Summary values for WHO anti-SARS-CoV-2 IgG (20/136)[8]

**Virus: SARS-CoV-2 D614G Variant**		**Calibration Factor**
Arithmetic mean ID50*	4135	0.242
Arithmetic mean ID80*	666	1.502
Median ID50	2422	0.413
Median ID80	489	2.045
Geometric Mean ID50	3047	0.328
Geometric Mean ID80	567	1.764

*As in Gilbert et al.[5], the arithmetic mean calibration factors were chosen to use for expressing the ID50 and ID80 results in IU50/ml and IU80/ml, respectively.

Table reproduced from the Supplementary Materials of Gilbert et al.[5] See pp. 23-24 of the Gilbert et al. supplement for further information on the validation experiment and calibration.

ID50, 50% inhibitory dilution; ID80, 80% inhibitory dilution; IU, International Unit.

Table S5. Meso-Discovery binding antibody assay and pseudovirus neutralization assay limits of the four antibody markers evaluated as immune correlates.

	Binding Antibody Assay		Pseudovirus Neutralizing Antibody Assay	
Reported Units	Binding Antibody Units (BAU)/ml*		International Units (IU): IU50/ml (ID50); IU80/ml (ID80)**	
	Spike	RBD	ID50	ID80
Positivity Cutoff	10.8424	14.0858	2.42	15.02
LOD	0.3076	1.5936	2.42	15.02
LLOQ	1.7968	3.43	4.477	21.4786
ULOQ	10,155.95	16,269	10919	15368

*Arbitrary units (AU)/ml converted to BAU/ml for all data analysis.

**Original titers calibrated to the WHO anti-SARS-CoV-2 immunoglobulin International Standard (NIBSC code: 20/136)[8] for all data analyses. ID50 = ID50 titer in IU50/ml; ID80 = ID80 titer in IU80/ml.

LOD, limit of detection; LLOQ, lower limit of quantitation; ULOQ, upper limit of quantitation.

Table S6. Cut-points defining High (above median) and Low (below median) for (A) D57 antibody marker subgroups and (B) D29 antibody marker subgroups

A.	D57 Antibody marker	High vs. Low Cut-point
	D57 Spike	2094.00 BAU/ml
	D57 RBD	3094.00 BAU/ml
	D57 ID50	219.00 IU50/ml
	D57 ID80	495.00 IU80/ml
**B.**	**D29 Antibody marker**	**High vs. Low Cut-point**
	D29 Spike	204.00 BAU/ml
	D29 RBD	204.00 BAU/ml
	D29 ID50	10.00 IU50/ml
	D29 ID80	24.00 IU80/ml

BAU, binding antibody units; IU, international units.

Supplementary Text

Assumptions needed for the Gilbert et al.[2] *binary principal surrogate evaluation method*

The method employed is the "NEH-CB" method described in Section 5.4 of Gilbert et *al.*[2], where NEH-CB refers to the set of assumptions that are made. These assumptions are SUTVA (Stable Unit Treatment Value Assumption), ignorability (randomization to vaccine vs. placebo), loss to follow-up for COVID-19 is random right-censoring, and whether the antibody marker is measured is missing at random. In addition, the method assumes No Early Harm (NEH), which states that there are no individuals who would have a COVID-19 outcome before the marker was measured under assignment to vaccine but not under assignment to placebo. The method also assumes no variability in marker measurements of participants assigned to placebo (so-called 'Constant Biomarker' (CB) scenario), which holds for the COVE application given that the analysis restricts to individuals with negative SARS-CoV-2 diagnostic testing results at enrollment. Moreover, the method requires that users specify the values of three fixed sensitivity parameters β_2, β_3, and β_4 that are defined in Section 5.4. of Gilbert et *al.*[2] Now, Gilbert et *al.*[2] has an incorrect statement in Section 5.4 that a fourth sensitivity parameter β_0 is also required; this sensitivity parameter is only relevant if the immune marker under study has variability among placebo arm participants, which is not applicable for the CB scenario present for the NEH-CB method. The estimation method under NEH-CB is correctly described in Gilbert et *al.*[2], which does not make use of β_0. We define the three sensitivity parameters, and describe their interpretations. Let Y(1) and Y(0) be potential outcomes indicating whether COVID-19 occurs during follow-up if assigned vaccine or placebo, respectively. Let $Y^{\tau}(1)$ and $Y^{\tau}(0)$ be potential outcomes indicating whether COVID-19 occurs by the time point τ at which the immune marker is measured (D29 or D57) if assigned vaccine or placebo, respectively. Let S(1) indicate the marker value at the time point of interest (D29 or D57) if assigned vaccine. With this notation, the sensitivity parameters β_2, β_3, and β_4 are defined based on specified pattern mixture models **B.2**, **B.3**, **B.4**, respectively:

B.2 $\exp(\beta_2) = [\text{risk1}(0, 0)/\{1-\text{risk1}(0, 0)\}] \div [\text{risk1}(0, *)/\{1-\text{risk1}(0, *)\}]$

B.3 $\exp(\beta_3) = [\text{risk1}(1, 0)/\{1-\text{risk1}(1, 0)\}] \div [\text{risk1}(1, *)/\{1-\text{risk1}(1, *)\}]$

B.4 $\exp(\beta_4) = [P(S(1)=1|0, 0)/\{1-P(S(1)=1|0, 0)\}] \div [P(S(1)=1|0, 1)/\{1-P(S(1)=1|0, 1)\}]$,

where $\text{risk1}(s_1, 0) \equiv P(Y(1) = 1|S(1) = s_1, S(0) = 0, Y^\tau(1) = 0, Y^\tau(0) = 0)$, $\text{risk1}(s_1, *) \equiv P(Y(1) = 1|S(1) = s_1, S(0) = *, Y^\tau(1) = 0, Y^\tau(0) = 1)$ for $s_1 = 0, 1$, and $P(S(1) = 1|0, y) = P(S(1) = 1| Y^\tau(1) = 0, Y^\tau(0) = y)$ for $y = 0, 1$. Here * indicates that the marker $S(0)$ is undefined because $Y^\tau(0) = 1$ (the marker is only defined if a participant is free of the disease outcome by time τ).

We interpret each sensitivity parameter for an analysis that measures an immune marker at the D57 visit; the parallel interpretation holds for an immune marker measured at the D29 visit. To intepret β_2, note that under the NEH assumption, a vaccine recipient remaining early-at-risk by τ and with marker value of 1 ($Y^\tau(1) = 0$ and $S(1) = 1$) can belong to either one of the two strata defined by counterfactual variables if assigned placebo: remaining early-at-risk by τ (and hence automaticaly with marker value $S(0) = 0$) and experiencing disease by τ (and hence with $S(0)$ undefined, $S(0) = *$). For vaccine recipients remaining early-at-risk and with Low marker value $S(1) = 0$, **B.2** defines a pattern mixture model for the two strata, with β_2 the log odds ratio of disease under placebo assignment and being early-at-risk ($Y^\tau(0) = 0$) relative to placebo assignment and experiencing early-disease ($Y^\tau(0) = 1$). The concept is that the fact of experiencing early-disease if assigned placebo may be correlated with unmeasured prognostic factors, and β_2 expresses a particular way to quantify this hidden correlation. The parameter β_3 is interpreted in the same way as for β_2, except for vaccine recipients with High marker value $S(1) = 1$. To intepret β_4, a vaccine recipient remaining early-at-risk by τ ($Y^\tau(1) = 0$) can belong to either one of two strata defined by counterfactual variables if assigned placebo: remaining early-at-risk by τ and experiencing disease by τ. These two strata are the same as for defining β_2 and β_3 except they do not involve the marker variable $S(1)$. For vaccine recipients remaining early-at-risk, **B.4** defines a pattern mixture model for the two

strata, with β_4 the log odds ratio of High marker value S(1) = 1 under placebo assignment and being early-at-risk ($Y^{\tau}(0) = 0$) relative to placebo assignment and experiencing early-disease ($Y^{\tau}(0) = 1$).

Supplementary References

1. Hejazi NS, van der Laan MJ, Janes HE, Gilbert PB, Benkeser DC. Efficient nonparametric inference on the effects of stochastic interventions under two-phase sampling, with applications to vaccine efficacy trials. *Biometrics* 2021; **77**(4): 1241-53.
2. Gilbert PB, Blette BS, Shepherd BE, Hudgens MG. Post-randomization Biomarker Effect Modification Analysis in an HIV Vaccine Clinical Trial. *J Causal Inference* 2020; **8**(1): 54-69.
3. Huang Y, Zhuang YY, Gilbert P. Sensitivity Analysis for Evaluating Principal Surrogate Endpoints Relaxing the Equal Early Clinical Risk Assumption. *Ann Appl Stat* 2022; **16**(3): 1774-94.
4. Benkeser D, Montefiori DC, McDermott AB, et al. Comparing antibody assays as correlates of protection against COVID-19 in the COVE mRNA-1273 vaccine efficacy trial *Science Translational Medicine* 2023; **15**(692): eade9078.
5. Gilbert PB, Montefiori DC, McDermott AB, et al. Immune correlates analysis of the mRNA-1273 COVID-19 vaccine efficacy clinical trial. *Science* 2022; **375**(6576): 43-50.
6. Baden LR, El Sahly HM, Essink B, et al. Efficacy and Safety of the mRNA-1273 SARS-CoV-2 Vaccine. *N Engl J Med* 2021; **384**(5): 403-16.
7. El Sahly HM, Baden LR, Essink B, et al. Efficacy of the mRNA-1273 SARS-CoV-2 Vaccine at Completion of Blinded Phase. *N Engl J Med* 2021; **385**(19): 1774-85.
8. National Institute for Biological Standards and Control (NIBSC). Instructions for use of First WHO International Standard for anti-SARS-CoV-2 Immunoglobulin (Version 3.0, Dated 17/12/2020) NIBSC code: 20/136 https://www.nibsc.org/science_and_research/idd/cfar/covid-19_reagents.aspx Access date Jul 29, 2021.
